# The Na_V_1.5 auxiliary subunit FGF13 modulates channels by regulating membrane cholesterol independent of channel binding

**DOI:** 10.1172/JCI191773

**Published:** 2025-08-12

**Authors:** Aravind R. Gade, Mattia Malvezzi, Lala Tanmoy Das, Maiko Matsui, Cheng-I J. Ma, Keon Mazdisnian, Steven O. Marx, Frederick R. Maxfield, Geoffrey S. Pitt

**Affiliations:** 1 Cardiovascular Research Institute, Weill Cornell Medicine, New York, New York, USA.; 2 Tri-Institutional Weill Cornell/Rockefeller/Sloan Kettering Medical Scientist Training Program, New York, New York, USA.; 3 Department of Biochemistry, Weill Cornell Medicine, New York, New York, USA.; 4 Division of Cardiology, Department of Medicine, and Department of Pharmacology, Vagelos College of Physicians and Surgeons, Columbia University, New York, New York, USA.

**Keywords:** Cardiology, Neuroscience, Cholesterol, Ion channels, Sodium channels

## Abstract

Fibroblast growth factor homologous factors (FHFs) bind to the cytoplasmic C-terminus of voltage-gated sodium channels (VGSCs) and modulate channel function. Variants in FHFs or VGSCs perturbing that bimolecular interaction are associated with arrhythmias. Like some channel auxiliary subunits, FHFs exert additional cellular regulatory roles, but whether these alternative roles affect VGSC regulation is unknown. Using a separation-of-function strategy, we show that a structurally guided, binding-incompetent, mutant fibroblast growth factor 13 (FGF13; the major FHF in mouse heart), confers complete regulation of VGSC steady-state inactivation (SSI), the canonical effect of FHFs. In cardiomyocytes isolated from *Fgf13-*KO mice, expression of the mutant FGF13 completely restores WT regulation of SSI. FGF13 regulation of SSI derives from effects on local accessible membrane cholesterol, which is unexpectedly polarized and concentrated in cardiomyocytes at the intercalated disc (ID), where most VGSCs localize. *Fgf13*-KO eliminates the polarized cholesterol distribution and causes loss of VGSCs from the ID. Moreover, we show that the previously described FGF13-dependent stabilization of VGSC currents at elevated temperatures depends on the cholesterol mechanism. These results provide new insights into how FHFs affect VGSCs and alter the canonical model by which channel auxiliary subunits exert influence.

## Abstract 2

The cardiac voltage-gated sodium channel auxiliary subunit Na_V_1.5 regulates the channel by binding dependent and binding-independent mechanisms.

## Graphical abstract

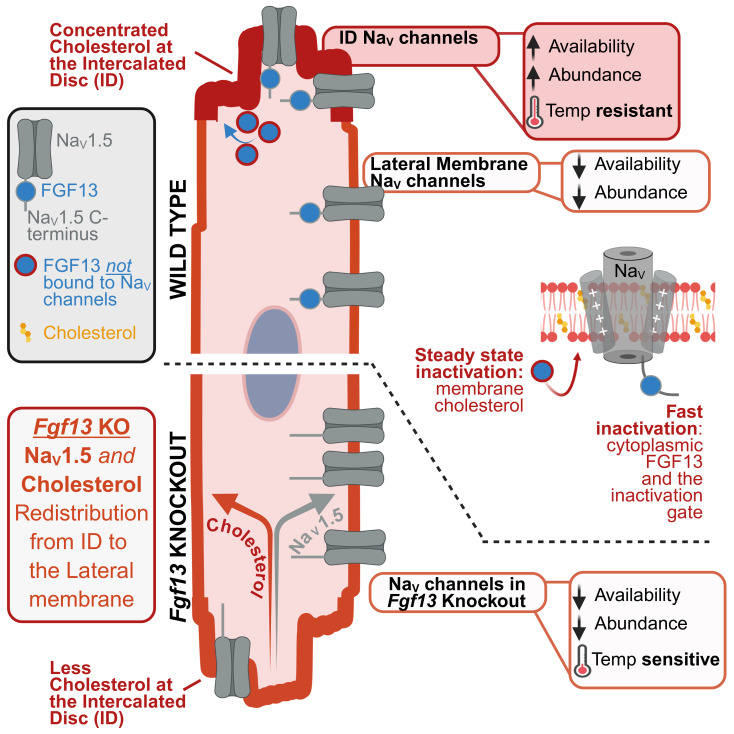

## Introduction

While pore-forming subunits of ion channels are responsible for ion conduction, auxiliary subunits critically influence channel function, as reflected by variants in multiple auxiliary subunit genes that are associated with arrhythmia syndromes (1, 2). Among their roles, auxiliary subunits control trafficking of channels to the sarcolemma, target channels to specific subcellular locations on the membrane, and tune biophysical properties of the channels inserted into the membrane. Auxiliary subunits are thought to affect channel function via direct binding mechanisms. This direct interaction model, however, is incomplete as some ion channel auxiliary subunits only “moonlight” as channel auxiliary subunits while serving other essential roles independent of channel interaction. A prime example is calmodulin, the most abundant intracellular Ca^2+^ binding protein and a regulator of numerous Ca^2+^ signaling events. Calmodulin also binds directly to, and serves as an auxiliary subunit for, multiple channels such as voltage-gated sodium channels (VGSCs), Ca^2+^ channels, and Κ^+^ channels (3–12). Whether the other cellular functions controlled by these auxiliary subunits affect channel function, however, is underexplored.

Here, we focus on the fibroblast growth factor homologous factor (FHF) subfamily of fibroblast growth factors (FGFs). The FHFs (FGF11–FGF14) comprise a noncanonical FGF subset that is not secreted (13, 14) and does not function as growth factors (15). FHFs can bind to the cytoplasmic VGSC C-termini (16) and modulate various aspects of VGSC function, one of the most prominent of which is increasing channel availability (i.e., a depolarizing shift in the half-maximal voltage [V_1/2_] of steady-state inactivation [SSI]) (17–19). Among the FHFs, human cardiomyocytes express predominantly *FGF12* and to a lesser extent *FGF13* (19–21). Variants of *FGF12* have been associated with inherited ventricular arrhythmias (20, 22), including Brugada syndrome (BrS), an arrhythmia characterized by reduced Na_V_1.5 (the major cardiac VGSC, encoded by *SCN5A*) current. A GWAS identified *FGF13* as a risk locus for atrial fibrillation (AF) (23), and postoperative AF is associated with reduced *FGF13* expression (24). Rodents predominantly express *Fgf13* in the ventricle (19) and cardiomyocyte-restricted *Fgf13* knockdown or KO affects VGSC currents (19, 25–28). The most consistent observed effect in *Fgf13-*KO mice — present in all studies to date (19, 25–28) — is a decrease in channel availability. Further, in *Fgf13-*KO cardiomyocytes, elevated temperature slows conduction velocity through the ventricular myocardium (26), a feature associated with BrS, in which affected individuals are at approximately 20-fold increased risk of a type I BrS ECG pattern and arrhythmias in the setting of a fever (29). How FGF13 protects against the effects of elevated temperature in the myocardium or how reduced FGF13 contributes to postoperative AF in humans is not known, thus preventing development of targeted therapies.

The working hypothesis for how FHFs regulate VGSCs is by a structurally defined direct interaction between the FHF and the cytoplasmic VGSC C-terminus via binding determinants within FHFs and VGSCs that are conserved among their respective family members (30, 31). A mutation within the cardiac VGSC Na_V_1.5 C-terminus that inhibits FHF binding is associated with ventricular arrhythmias and sudden cardiac death in a 5-generation family (32), underscoring the importance of this interaction. Similarly, a mutation perturbing the corresponding interaction site on FGF12, an FHF expressed in human brain and heart, affects neuronal VGSC function (33) and is associated with a seizure disorder (34). This mutation also affects cardiac VGSC currents when the mutant FGF12 is expressed in rodent cardiomyocytes (33). A separate variant in *FGF12* that impairs FGF12 interaction with Na_V_1.5 has been associated with BrS (20). FHFs are evolutionarily derived from FGFs and thus likely have multiple effects beyond direct binding to VGSCs, and these other roles could affect VGSCs in a binding-independent manner. Indeed, FGF13 stabilizes microtubules (14, 35, 36), which are critical for trafficking and targeting VGSCs (37).

Here, using a structurally informed FGF13 mutant unable to bind VGSCs, we show that FGF13 exerts critical components of its VGSC regulatory functions, including the canonical shift in SSI, via binding-independent mechanisms.

## Results

**Figure 1:**
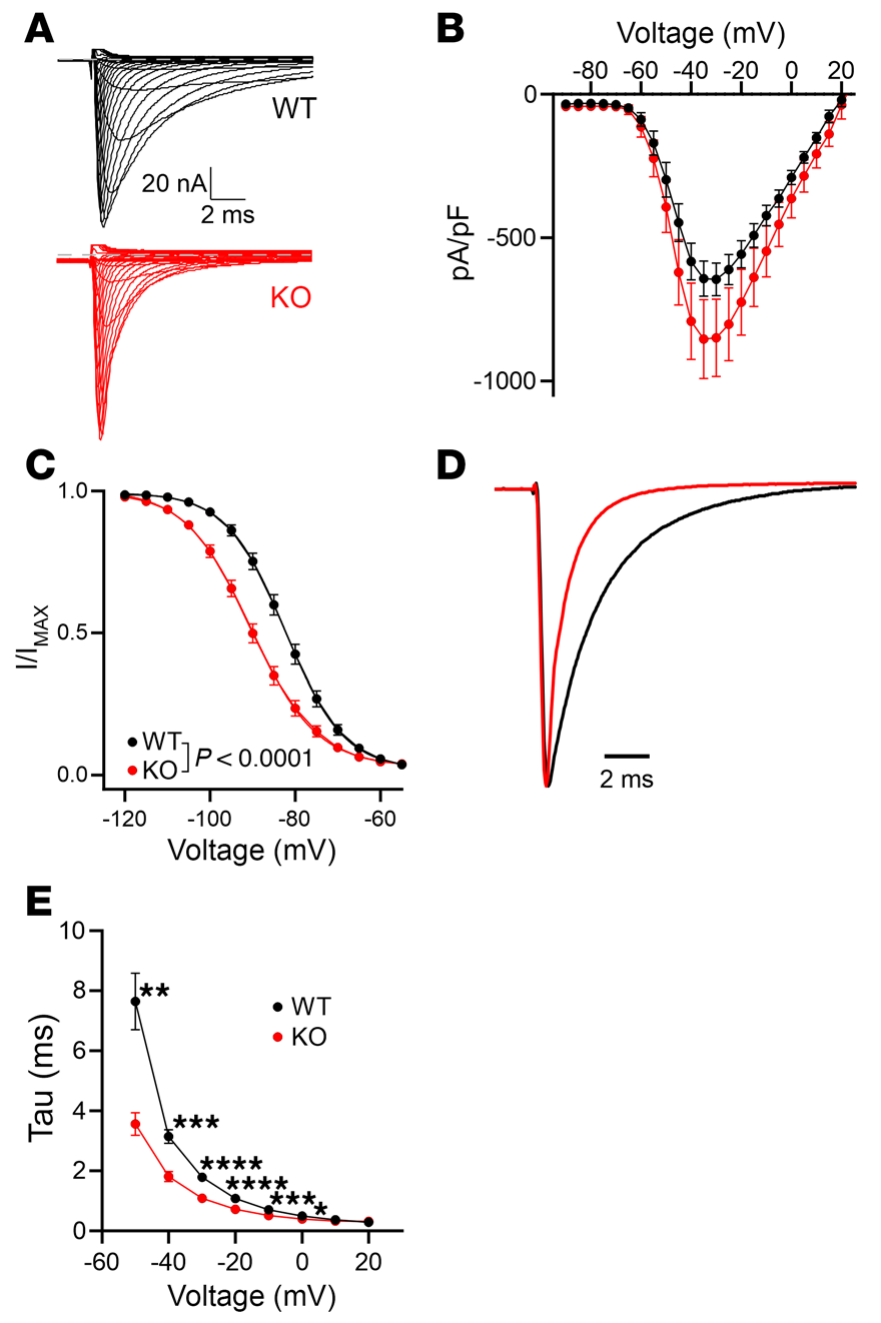
FGF13 regulates VGSC kinetics in cardiomyocytes. (**A**) Representative raw traces of sodium channel currents recorded from acutely isolated cardiac myocytes in response to depolarizing voltage steps from a holding potential of –120 mV. (**B**) Current-voltage relationship displaying peak current densities at various depolarizing voltages. (**C**) SSI curves showing normalized sodium currents elicited at –20 mV after conditioning from different holding potentials. (**D**) Representative raw traces of sodium currents at –20 mV, highlighting differences in fast inactivation kinetics. (**E**) Analyzed time constant of inactivation (τ) for sodium currents recorded at different voltages. Statistical analysis: 2-way ANOVA with Bonferroni’s post hoc test (*P < 0.05, **P < 0.01, ***P < 0.001, ****P < 0.0001).

We employed a constitutive cardiac-specific KO model (c*Fgf13^KO^*) to investigate how FGF13 regulates VGSCs in cardiomyocytes (38). Current amplitude was not different between genotypes (Figure 1, A and B, and Supplemental Table 1; supplemental material available online with this article; https://doi.org/10.1172/JCI191773DS1). As previously observed in an independent constitutive cardiac-specific KO model (27), an inducible KO model (25), a *Fgf13* hypomorphic line (26), and after *Fgf13* knockdown (19, 28), elimination of FGF13 hyperpolarized the V_1/2_ of VGSC SSI recorded from acutely isolated cardiomyocytes (V_1/2_ for WT, –82.4 ± 0.8 mV vs. –90.4 ± 0.9 mV for c*Fgf13^KO^*; Figure 1C and Supplemental Table 1). Also as previously reported (27), FGF13 ablation accelerated the rate of VGSC fast inactivation (τ; Figure 1, D and E, and Supplemental Table 1). Since SSI depends primarily on the actions of membrane-delimited voltage sensors, we investigated how FHFs restricted to the cytoplasm affect this property and considered the possibility that FGF13 exerted effects on VGSCs independent of FGF13’s interaction with Na_V_1.5.

**Figure 2:**
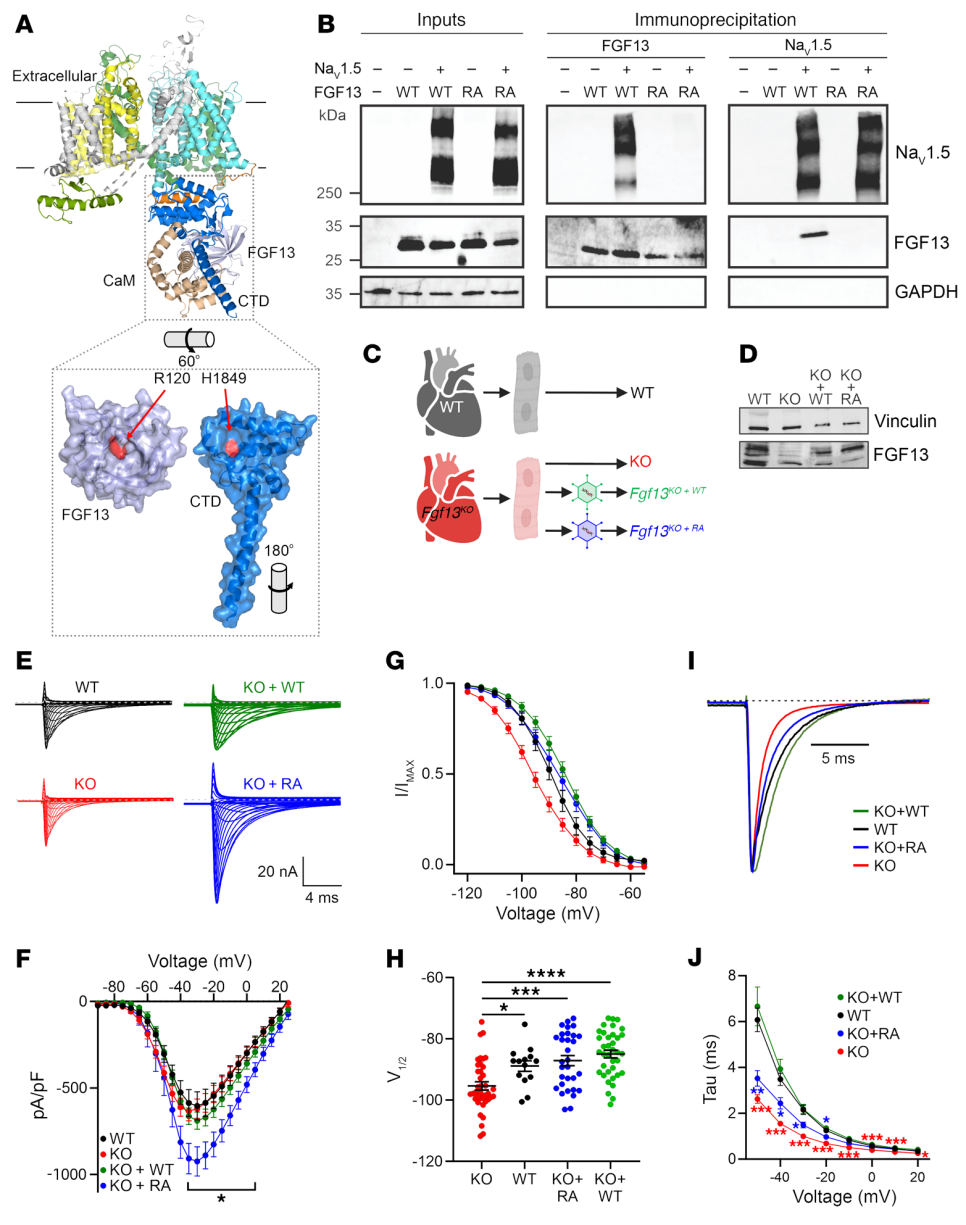
A binding-incompetent FGF13 mutant confers a subset of WT-like regulatory functions in cardiomyocytes. (**A**) Overlay of the insect sodium channel crystal structure (PDB: 5X0M) with the Nav1.5 C-terminal domain (CTD) in complex with FGF13 (PDB: 4DCK), illustrating the FGF13 binding site on Na_V_1.5 CTD. (**B**) Immunoprecipitation of FGF13 or Na_V_1.5 in HEK cells coexpressing Nav1.5 and either WT or mutant (R120A) FGF13 (FGF13^R/A^), showing a loss of binding between FGF13^R/A^ and Na_V_1.5 (representative example from 3 independent experiments). (**C**) Experimental strategy to re-express FGF13 or FGF13^R/A^ in cardiac myocytes from Fgf13-KO mice using adeno-associated virus vectors. (**D**) Western blot confirming the expression of FGF13 and FGF13^R/A^ in KO myocytes after rescue. (**E**) Representative sodium current traces from all experimental groups. (**F**) Current-voltage relationships showing peak current densities. (**G**) Normalized SSI curves. (**H**) V_1/2_ of inactivation showing rescue effects of FGF13 and FGF13^R/A^. (**I**) Representative sodium current traces at –20 mV (normalized to peak current) illustrating differences in fast inactivation. (**J**) Time constants (τ) of inactivation at varying voltages. Statistical analysis: 2-way ANOVA with Bonferroni’s post hoc test (*P < 0.05, **P < 0.01, ***P < 0.001, ****P < 0.0001).

To test our hypothesis, we exploited previous structural studies (30, 31) and developed a strategy to replace FGF13 in cardiomyocytes with a mutant version incapable of binding to Na_V_1.5. The binding-incompetent FGF13 bears an alanine substitution at Arg^120^ (FGF13^R/A^; Figure 2A), thus eliminating a side chain that inserts into a deep hole on the Na_V_1.5 C-terminal domain surface with the channel’s His^1849^ at the hole’s base. Both residues are conserved among FHFs and VGSCs, respectively (Supplemental Figure 1), and are critical for FHF–VGSC interaction (30): variants in either the FHF Arg or the Na_V_1.5 His residue disrupt binding and are associated with disorders characterized by abnormal VGSC function (32, 39). For these FGF13 expressing viruses, and in additional heterologous expression system studies, we used exclusively the Fgf13-VY splice variant, which is the dominant *Fgf13* transcript expressed in ventricles. Transcripts for the major neuronal splice variant, *Fgf13-S*, are <<10% of *Fgf13-VY*, and FGF13-S protein is not detected in Western blots of mouse ventricle (19). We confirmed that FGF13^R/A^ eliminated interaction with Na_V_1.5 in HEK293 cells by expressing FGF13 or FGF13^R/A^ with Na_V_1.5 and performing immunoprecipitation of FGF13. Figure 2B shows that Na_V_1.5 coimmunoprecipitated with FGF13, but not FGF13^R/A^ (example coimmunoprecipitation of 3 independent experiments), analogous to previous data (40) obtained with a homologous FGF14 mutant and Na_V_1.6. Moreover, in a previous study (Figure 6B in ref. 41), we specifically tested the interaction between FGF13^R/A^ and the Na_V_1.5 C-terminal domain by isothermal titration calorimetry and observed that the WT FGF13 bound Na_V_1.5 with *K_D_* = 123 ± 2 nM and the R/A mutation completely eliminated interaction. With these results, we were able to separate FGF13 regulation of VGSCs by direct interaction and by indirect means. We therefore virally expressed FGF13 or FGF13^R/A^ in ventricular cardiomyocytes isolated from c*Fgf13^KO^* for electrophysiological analyses and compared results with those obtained from cardiomyocytes isolated from WT mice or c*Fgf13^KO^* mice (Figure 2C). To allow for viral expression, recordings were performed 24–72 hours after isolation and immediate infection. In c*Fgf13^KO^* mice, we confirmed efficient KO of FGF13 and similar levels of expressed FGF13 or FGF13^R/A^ protein from their respective viruses (Figure 2D). VGSC current density was larger in c*Fgf13^KO^* cardiomyocytes infected with the FGF13^R/A^ virus, but current densities in the other groups were indistinguishable from each other (Figure 2, E and F, and Supplemental Table 1).

We then analyzed SSI and kinetics of fast inactivation. As in acutely isolated cardiomyocytes, the V_1/2_ for SSI in WT was significantly depolarized compared with the V_1/2_ for SSI in c*Fgf13^KO^* cardiomyocytes (Figure 2, G and H, and Supplemental Table 1). Expression of FGF13 in c*Fgf13^KO^* cardiomyocytes significantly depolarized the V_1/2_ of SSI, demonstrating successful rescue. Similarly, re-expression of the binding-incompetent FGF13^R/A^ depolarized the V_1/2_ of SSI (Figure 2, G and H), suggesting that FGF13 binding to Na_V_1.5 is not necessary for regulation of SSI. Also as in acutely isolated cardiomyocytes, VGSC currents in cultured c*Fgf13^KO^* cardiomyocytes showed faster inactivation than in WT cardiomyocytes. Viral expression of FGF13 in c*Fgf13^KO^* cardiomyocytes slowed VGSC inactivation, but viral expression of FGF13^R/A^ did not (Figure 2, I and J, and Supplemental Table 1). Thus, fast inactivation requires interaction between FGF13 and the channel’s C-terminal domain. We obtained concordant results with a heterologous expression system. In HEK293 cells, we expressed Na_V_1.5 with FGF13 (either WT or FGF13^R/A^) and GFP, or with GFP only. Expression of Na_V_1.5 with GFP only is analogous to *Fgf13^KO^* in cardiomyocytes since HEK293 cells do not express endogenous FGF13, while expression of Na_V_1.5 with FGF13 corresponds to WT cardiomyocytes. As in cardiomyocytes, Na_V_1.5 SSI was hyperpolarized and τ was faster in the absence of FGF13 (GFP expression only) than with FGF13. Also as in cardiomyocytes, expression of the binding-incompetent FGF13^R/A^ restored SSI but not τ (Supplemental Figure 2, A–E).

How does FGF13 affect Na_V_1.5 SSI via a binding-independent mechanism? Since SSI is generally regulated by the membrane-delimited voltage sensors and — having excluded a direct interaction mechanism — a hyperpolarizing shift in VGSC SSI is near-pathognomonic for a reduction in membrane stiffness via depletion of accessible membrane cholesterol (42–44), we considered biochemical data showing that FGF13 regulates local membrane cholesterol content through interaction with cavins (45), a set of caveolae regulator proteins (46), without affecting the total cholesterol pool. Specifically, we hypothesized that FGF13 affected local membrane cholesterol, independent of binding to Na_V_1.5, to regulate SSI of Na_V_1.5 in cardiomyocytes. As cholesterol regulation of VGSC gating may be channel specific, as suggested by differing effects after cholesterol depletion (42–44, 47), we first tested whether manipulation of membrane cholesterol affected Na_V_1.5 channels. Indeed, depletion of membrane cholesterol with methyl-β-cyclodextrin (MβCD) induced a hyperpolarizing shift in the V_1/2_ of SSI for Na_V_1.5 (coexpressed with GFP as a control) and for Na_V_1.5 coexpressed with FGF13, albeit to a lesser extent (Supplemental Figure 3, A and B). In contrast, addition of cholesterol depolarized SSI for Na_V_1.5 (coexpressed with GFP) but did not affect SSI for Na_V_1.5 coexpressed with FGF13 (Supplemental Figure 3, C and D). Although not a focus of this study, we note that cholesterol depletion exerts distinct effects on activation of specific channels in different cell types (42–44, 47). We therefore tested whether and how MβCD affects the V_1/2_ of Na_V_1.5 activation. In HEK293 cells, cholesterol depletion with MβCD hyperpolarized the V_1/2_ of activation (Supplemental Figure 3, E and F), similar to observed effects on Na_V_1.9 channels (47). Coexpressing FGF13 yielded a V_1/2_ for activation not different from Na_V_1.5 alone, yet blocked the hyperpolarizing shift when MβCD was added.

**Figure 3:**
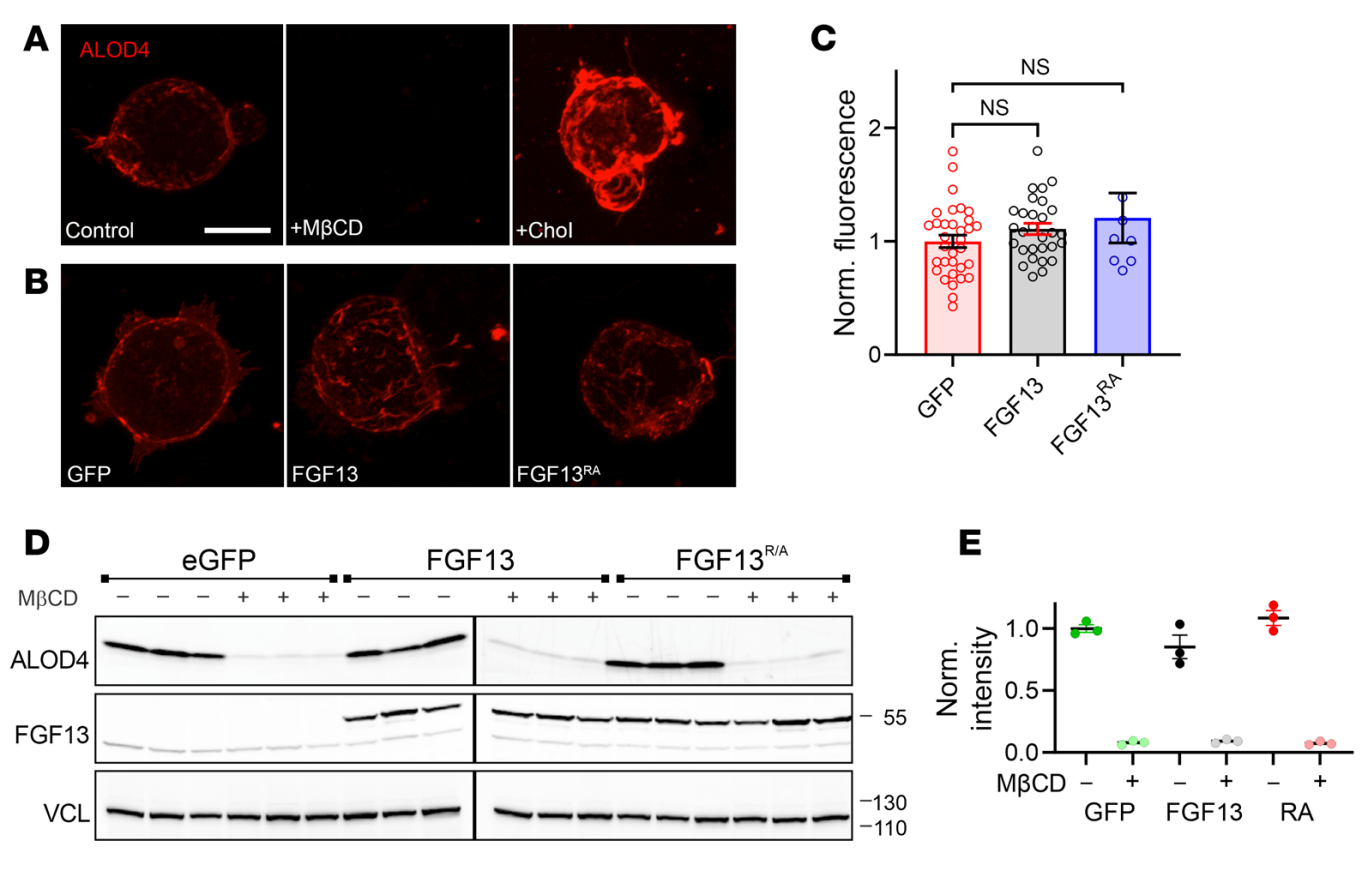
ALOD4 detects membrane-accessible cholesterol. (**A** and **B**) Confocal images of ALOD4 staining in HEK cells under control, MβCD-treated, and cholesterol-enriched conditions (scale bar: 10 μm) (**A**) and after expression of GFP, FGF13, and FGF13^R/A^ (**B**). (**C**) Quantification of ALOD4 intensity, showing no significant differences between FGF13 and FGF13^R/A^-expressing cells. Statistical analysis: 1-way ANOVA. (**D** and **E**) In-gel fluorescence imaging and quantification of ALOD4 showing no difference in total membrane-accessible cholesterol in HEK cells expressing FGF13, FGF13^R/A^, or GFP.

Furthermore, we showed that FGF13 affected membrane cholesterol by exploiting ALOD4, a recombinant domain of the soluble bacterial toxin anthrolysin O that binds accessible plasma membrane cholesterol (48, 49), to visualize effects in HEK293 cells. ALOD4 labels the small pool of membrane cholesterol (<10% of total) that is metabolically active (50) and most accessible to cholesterol-depleting reagents such as cyclodextrins. Application of ALOD4 to live cells labeled the plasma membrane–accessible cholesterol pool, as observed by fluorescent imaging of HEK293 cells (Figure 3A and Supplemental Figure 4A). Incubation with MβCD (500 μM for 60 minutes) depleted the ALOD4 fluorescent signal, while addition of cholesterol augmented the signal (Figure 3A and Supplemental Figure 4A), showing specificity of ALOD4. We then queried whether the presence of FGF13 or FGF13^R/A^ (compared with GFP control) affected the ALOD4 signal. In control GFP-transfected cells, the ALOD4 signal in maximum projection confocal stacks appeared evenly distributed to the cell periphery, but with FGF13 or FGF13^R/A^ transfection, we observed irregular signal scattered throughout the image, consistent with the presence of membrane ruffles (Figure 3B and Supplemental Figure 4, B and C), which are regulated by local changes in cholesterol (51). Despite the difference in the pattern of fluorescent signal, the integrated total signal was not different among the 3 groups (Figure 3C), reflecting expected cellular homeostatic mechanisms (52) and consistent with previous data showing that total cholesterol in heart lysates was unperturbed in *Fgf13*-KO mice despite a change in local membrane cholesterol distribution (45). Consistent with the microscopy images, we prepared cellular lysates and quantified ALOD4 protein by gel electrophoresis and in-gel imaging of the ALOD4 fluorescence signal. Figure 3, D and E, shows ALOD4 signal depletion by MβCD within all groups and demonstrates that the overall accessible cholesterol is unaffected by expression of FGF13 or FGF13^R/A^. Together, these data show that FGF13 does not affect total cell cholesterol, but it does alter the distribution (compared with no FGF13) of accessible cholesterol in the membrane, consistent with previous biochemical data (45). Moreover, as HEK293 cells do not express endogenous Na_V_1.5, these data (along with data showing similar effects with the Na_V_1.5 binding-incompetent FGF13^R/A^) suggest that the effects of FGF13 on membrane cholesterol are independent of Na_V_1.5 binding.

**Figure 4:**
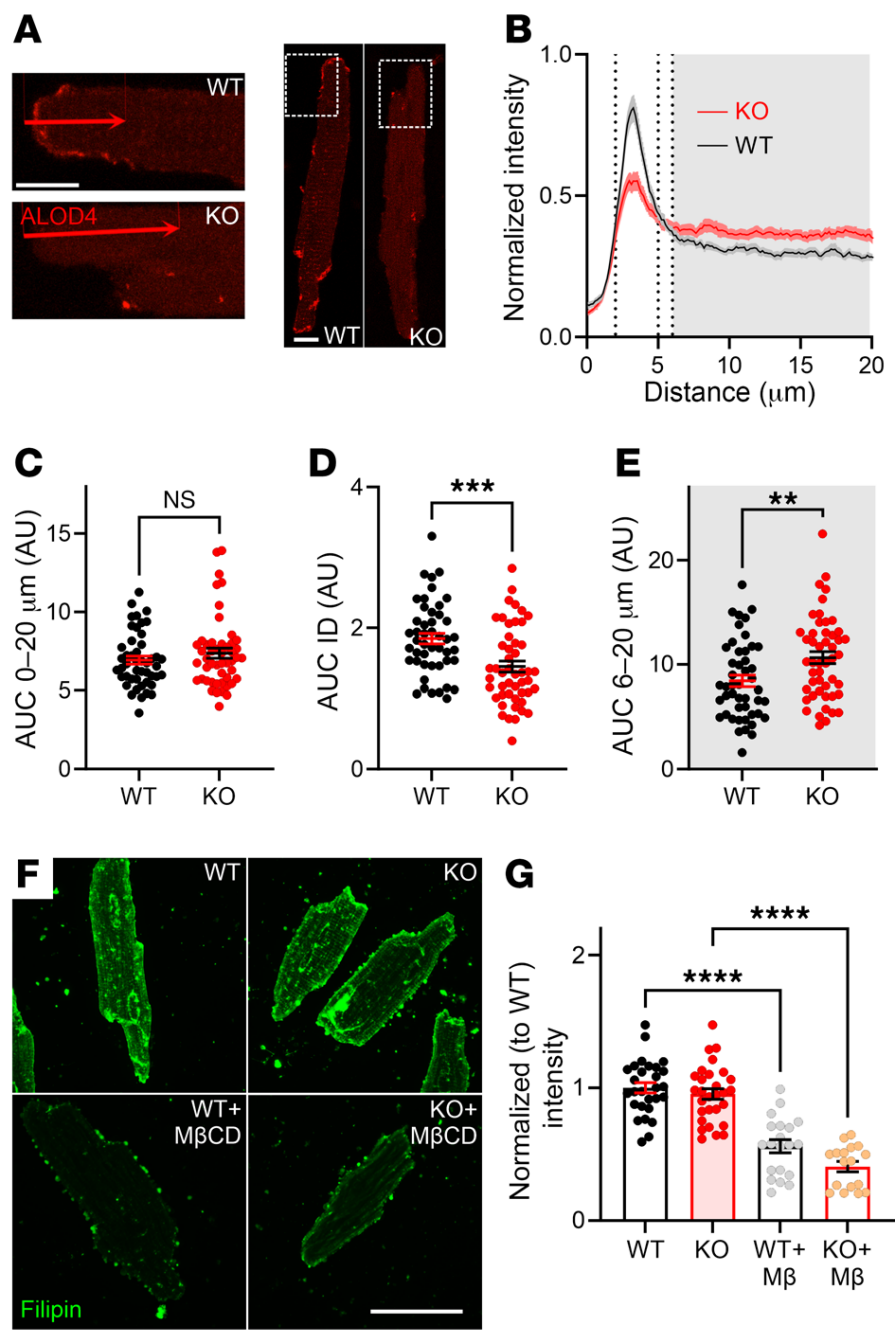
Accessible membrane cholesterol is concentrated at the IDs in cardiomyocytes and is regulated by FGF13. (**A**) Confocal images of ALOD4 staining in c*Fgf13^KO^* cardiac myocytes, showing altered distribution compared with the WT. Scale bar: 10 μm. (**B**) Quantification of ALOD4 signal from IDs to the lateral membrane, highlighting localization differences in c*Fgf13^KO^* myocytes. (**C**–**E**) Quantification of ALOD4 intensity distribution, showing no significant change in total signal intensity. Statistical analysis: unpaired *t* test (**P < 0.01, ***P < 0.001). (**F**) Filipin staining of cardiac myocytes, demonstrating overall cholesterol depletion following MβCD treatment. Scale bar: 50 μm. (**G**) Quantification of filipin signal intensity in WT and KO myocytes, showing no cholesterol differences. Statistical analysis: 1-way ANOVA with Bonferroni’s post hoc test (****P < 0.0001).

We then applied ALOD4 to isolated cardiomyocytes. In WT cells, accessible cholesterol is polarized, with concentration at the intercalated discs (IDs), as measured by line scans (starting 1 μm before the cell edge) perpendicular to the cardiomyocyte short axis (Figure 4, A–E, and Supplemental Figure 5A). In contrast, in cardiomyocytes from c*Fgf13^KO^* mice, the signal intensity from the ID was reduced (signal between 2 and 5 μm along the line), and relatively more signal was observed throughout the rest of the cell (measured between 6 and 20 μm along the line). We demonstrated the specificity of the ALOD4 signal in cardiomyocytes by showing that cholesterol depletion with MβCD reduced the signal in WT cardiomyocytes, while addition of cholesterol increased the signal (Supplemental Figure 5B). Despite the genotype differences in pattern and concentration of signal at the ID (Figure 4D), along 20 μm line scans there were no differences in AUC measurements (Figure 4C), suggesting that the total cholesterol was not different between genotypes. Indeed, quantifying total cholesterol with filipin fluorescence showed no difference (Figure 4, F and G). We confirmed the specificity of the filipin signal by showing its depletion after application of MβCD (Figure 4, F and G, and Supplemental Figure 5C). Together, these data showing that ablation of FGF13 drives redistribution of accessible membrane cholesterol from the cardiomyocyte ID to the midsection without affecting the total cholesterol pool are consistent with previous biochemical data showing that FGF13 ablation redistributes membrane cholesterol (45).

**Figure 5:**
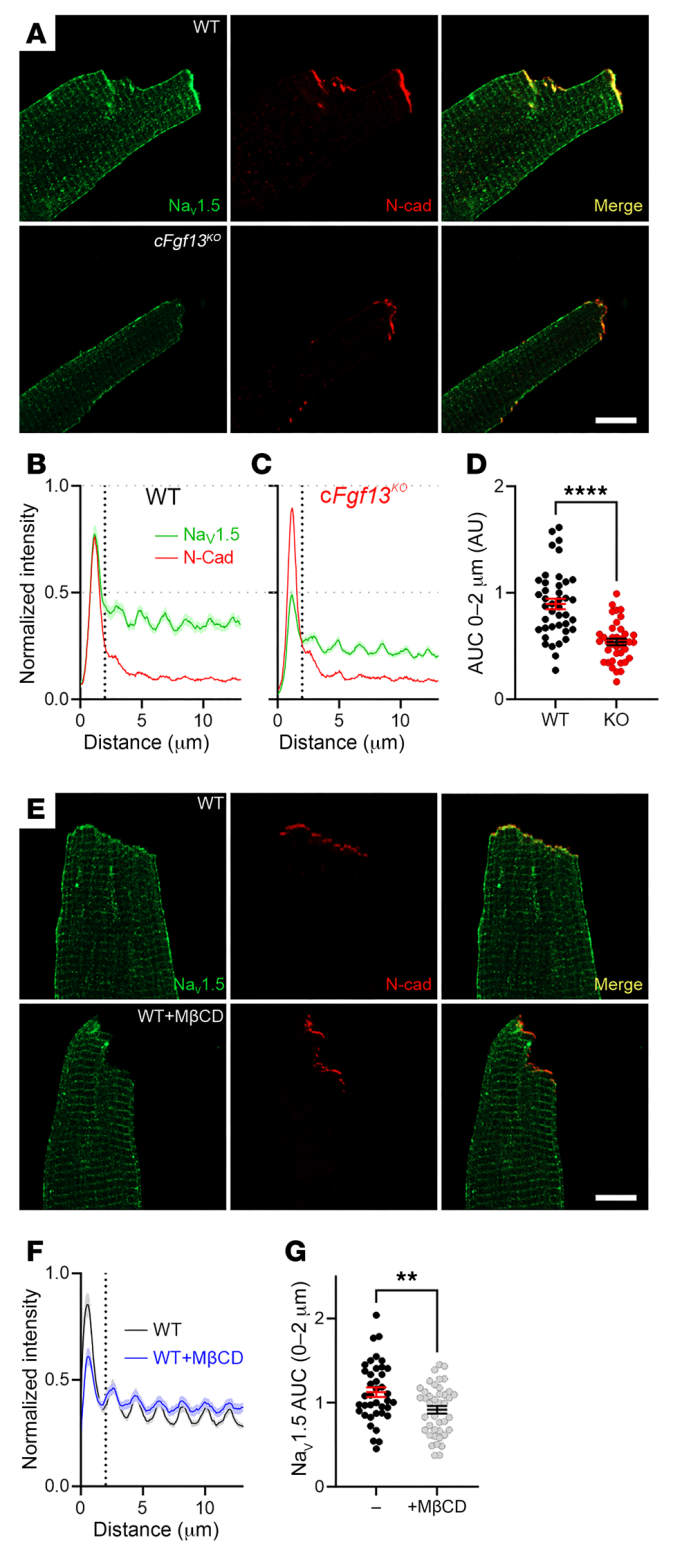
FGF13 and cholesterol regulate Na_V_1.5 at the ID. (**A**) Confocal images showing Nav1.5 localization in cardiac myocytes from WT and c*Fgf13^KO^* mice. Scale bar: 10 μm. (**B** and **C**) Quantification of Nav1.5 signal intensity at the ID and lateral membrane in WT and KO myocytes. (**D**) Total Nav1.5 signal intensity in KO myocytes, showing a significant reduction. Statistical analysis: unpaired *t* test (****P < 0.0001). (**E**) Confocal images of Nav1.5 in WT myocytes under control and MβCD-treated conditions. Scale bar: 10 μm. (**F** and **G**) Quantified Nav1.5 intensity at the ID and total levels after MβCD treatment, resembling the c*Fgf13^KO^* phenotype. Statistical analysis: unpaired *t* test (**P < 0.01).

Not only did *Fgf13*-KO affect membrane cholesterol distribution, but it also affected the amount of Na_V_1.5 at the ID. Figure 5A shows immunocytochemistry for Na_V_1.5 in cardiomyocytes isolated from WT or c*Fgf13^KO^* mice. Costaining with an antibody for N-cadherin (N-Cad) marked the ID, where 1 of the 2 major Na_V_1.5 channel pools resides. Fluorescence intensity line scans perpendicular to the cardiomyocyte short axis and normalized to the signal in WT cells showed a peak Na_V_1.5 signal in WT at the ID (overlapping with the N-Cad signal; Figure 5, A and B). The Na_V_1.5 fluorescence intensity in c*Fgf13^KO^* cells was 39.9% ± 14.6% lower (Figure 5C), as quantified by AUC measurements for the first 2 μm of the line scan (Figure 5D). We considered that the reduction in Na_V_1.5 at the ID in c*Fgf13^KO^* cells resulted from loss of FGF13-regulated trafficking of Na_V_1.5 to the ID, as previously hypothesized (45), and/or from reduction in local membrane cholesterol, as in Figure 4. To test if a reduction in local membrane cholesterol was sufficient to affect Na_V_1.5 at the IDs, we treated cardiomyocytes isolated from WT mice and observed that treatment with MβCD reduced the Na_V_1.5 fluorescence intensity at the ID by 18.6% ± 6.5% (Figure 5, E–G). Together, these data indicate that FGF13 ablation not only reduces Na_V_1.5 at the ID but that perturbation of membrane-accessible cholesterol by FGF13 ablation is a likely mechanism.

**Figure 6:**
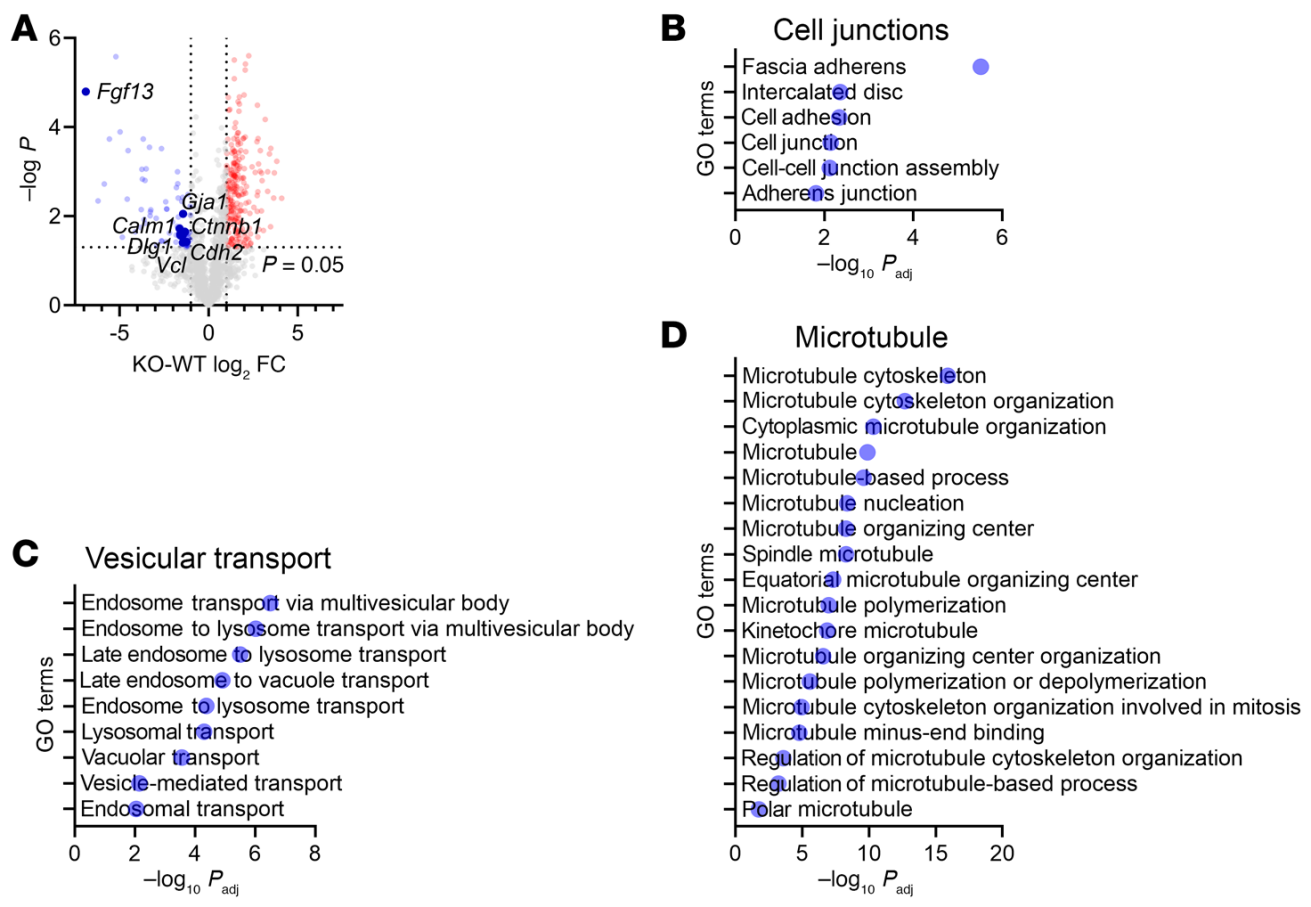
KO of *Fgf13* affects the localization of Na_V_1.5 at the ID. (**A**) Volcano plot of proteins coimmunoprecipitated with Na_V_1.5 from WT or c*Fgf13^KO^* hearts. Gene names for ID proteins are highlighted. (**B**–**D**) Gene set enrichment analyses of proteins enriched in coimmunoprecipitates of Na_V_1.5 in the WT (compared with c*Fgf13^KO^* hearts). GO, Gene Ontology.

Based on these imaging data, we hypothesized that known ID components would be depleted from Na_V_1.5 immunoprecipitates in c*Fgf13^KO^* hearts compared with WT hearts. We therefore identified by mass spectrometry proteins coimmunoprecipitated with Na_V_1.5 from WT or c*Fgf13^KO^* hearts (*n* = 3 each). Principal component analysis and Pearson’s correlation statistics showed that the set of proteins coimmunoprecipitated from WT hearts was distinct from the set coimmunoprecipitated from c*Fgf13^KO^* hearts (Supplemental Figure 6, A and B). We detected 74 coimmunoprecipitated proteins depleted (*P* < 0.05; log_2_FC ≥ 1.0) in KO hearts compared with WT hearts (Figure 6A and Supplemental Table 2); indeed, these proteins included several ID components (e.g., proteins encoded by *Gja1*, *Ctnnb1*, *Cdh2*, *Dlg1*, and *Vcl*). Furthermore, gene set enrichment analysis (Figure 6, B–D) showed that the top terms included fascia adherens (GO:0005916) and intercalated disc (GO:0014704), terms not found in the analysis of the set of coimmunoprecipitated proteins enriched in c*Fgf13^KO^* hearts (Supplemental Table 2). Also notable was the identification of calmodulin (*Calm1*) as one of the coimmunoprecipitated proteins depleted in KO hearts compared with WT hearts (log_2_FC = 1.63, *P* = 0.02) since a previous study reported that FGF13 increases the affinity between Na_V_1.5 and calmodulin (53). Moreover, 2 other sets of top Gene Ontology terms found in the analysis of coimmunoprecipitated proteins depleted from WT hearts were associated with vesicular transport and microtubules (Figure 6, C and D, and Supplemental Table 1). None of these terms were found in the analysis of the set of proteins enriched in the coimmunoprecipitates from c*Fgf13^KO^* hearts. Thus, these data underscore that FGF13 affects the subcellular distribution of Na_V_1.5 and provide mechanistic insight into the altered Na_V_1.5 subcellular localization observed in c*Fgf13^KO^* hearts.

**Figure 7:**
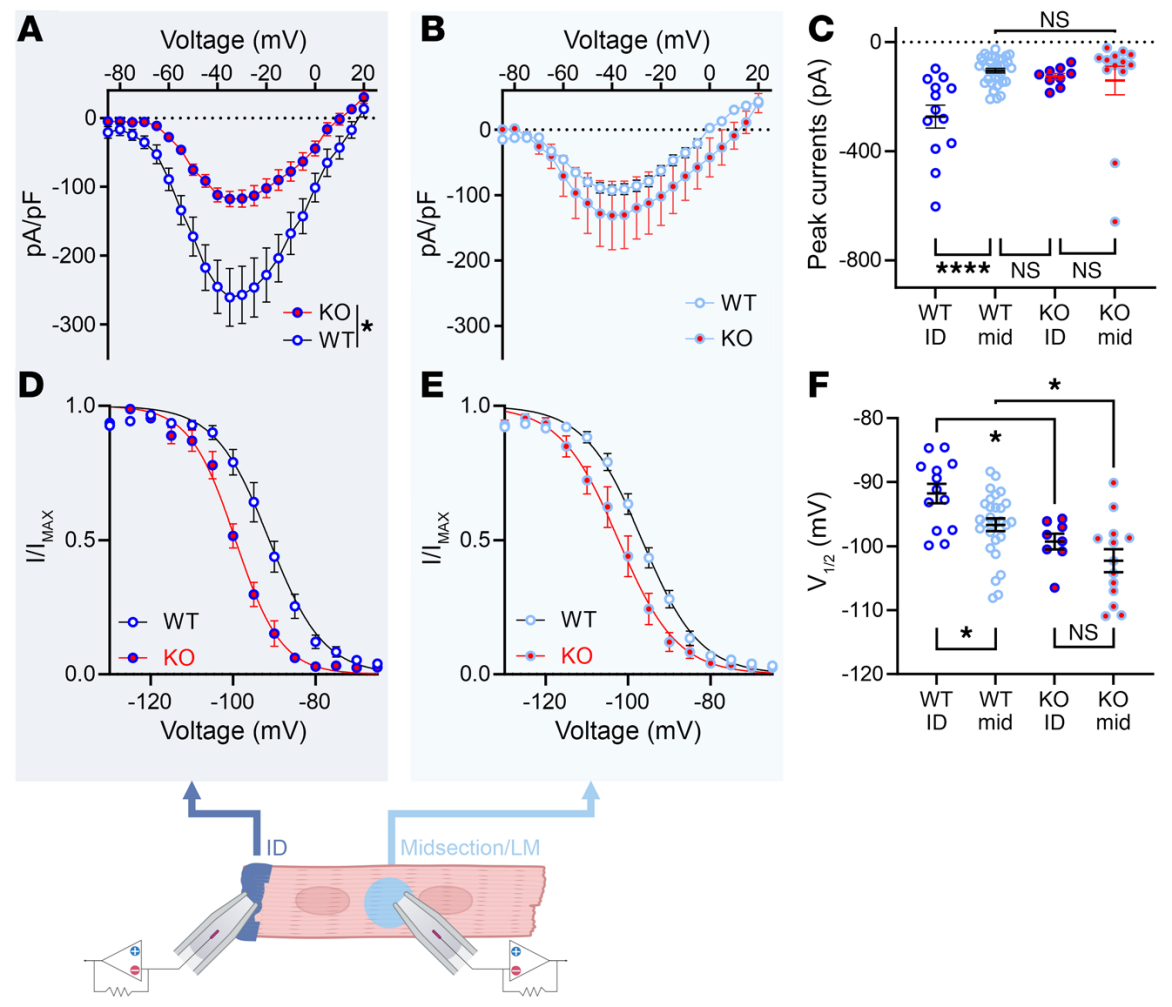
Macropatch experiments show loss of VGSC currents at the ID of cardiomyocytes from c*Fgf13^KO^* hearts. (**A** and **B**) Current-voltage relation curves from IDs (**A**) and lateral membranes (LM) (**B**) of WT and KO myocytes. (**C**) Peak sodium current densities showing differences between IDs and LMs of WT but not KO myocytes. Statistical analysis: 1-way ANOVA with Bonferroni’s post hoc test (****P < 0.0001). (**D** and **E**) SSI curves from IDs (**D**) and LM (**E**) of WT and KO myocytes. (**F**) V_1/2_ of inactivation values, showing significant differences between WT and KO myocytes at IDs (**D**) and LMs (**E**) and significant differences between IDs and LMs of WT but not KO myocytes. Statistical analysis: unpaired t test (*P < 0.05).

The redistribution of Na_V_1.5 away from the ID in the absence of FGF13 could affect VGSC properties based on a previous study showing differences in VGSC amplitude and SSI depending on where on the cardiomyocyte recordings were performed (54). Currents recorded from the ID were larger and the V_1/2_ of SSI was depolarized compared with currents recorded from the midsection (lateral membrane). We hypothesized that the change in subcellular distribution of Na_V_1.5 as regulated by FGF13 was an important contributor. We therefore performed macropatch recordings from the ID and midsection, which differed in WT versus c*Fgf13^KO^* cardiomyocytes. In WT cardiomyocytes, peak currents were larger at the ID than at the midsection, as expected, yet that differential was lost in c*Fgf13^KO^* cells and the current amplitudes at either the ID or midsection were not different from the midsection in WT cells (Figure 7, A–C, and Supplemental Table 1). The macropatch data also suggest that FGF13 contributes to the previously observed depolarization of the V_1/2_ of SSI at the ID compared with the midsection. In WT cells, SSI at the ID was depolarized compared with V_1/2_ at the midsection, but in c*Fgf13^KO^* cardiomyocytes, there was no statistically significant difference (Figure 7, D–F, and Supplemental Table 1). Using sensitivity to tetrodotoxin (TTX), the previous study suggested that the amplitude and SSI differences between the ID and midsection reflected predominantly TTX-resistant Na_V_1.5 channels expressed at the ID and TTX-sensitive neuronal VGSCs at the midsection (54). Here, we found that SSI of VGSCs at the ID and the midsection in WT cells was depolarized compared with their counterparts in c*Fgf13^KO^* cardiomyocytes. Thus, FGF13 affects SSI of VGSCs independent of channel location, suggesting that FGF13 affects both Na_V_1.5 and neuronal VGSCs expressed in cardiomyocytes (Supplemental Figure 2 and Supplemental Table 1).

**Figure 8:**
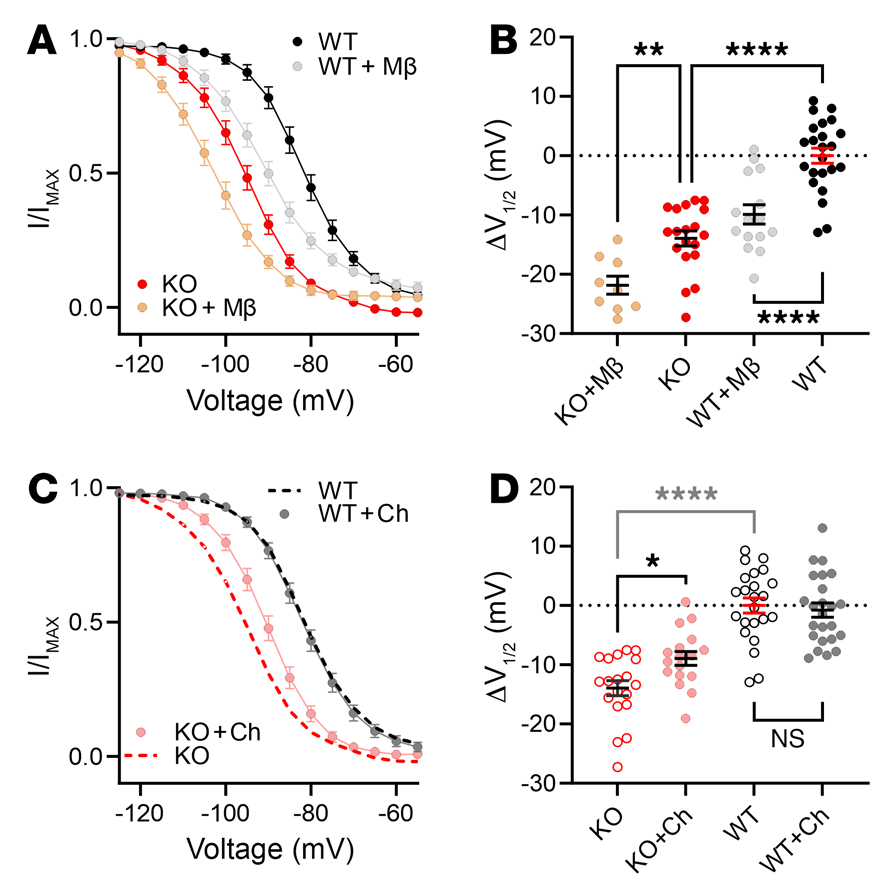
FGF13 differentially affects consequences of manipulation of membrane-accessible cholesterol on SSI of VGSCs in cardiomyocytes. (**A** and **C**) SSI curves of sodium currents from WT and KO myocytes under MβCD-treated (**A**) and cholesterol-enriched (**C**) conditions. (**B** and **D**) Changes in the V_1/2_ of inactivation values showing effects of MβCD treatment (**B**) and cholesterol enrichment (**D**). Data for WT and KO are repeated in **C** and **D**. Statistical analysis: 1-way ANOVA with Bonferroni’s post hoc test (*P < 0.05, **P < 0.01, ****P < 0.0001).

Since *Fgf13* ablation affects the polarized distribution of membrane-accessible cholesterol, which regulates SSI in HEK293 cells (Supplemental Figure 3 and Supplemental Table 1), we hypothesized that the lack of difference between the V_1/2_ of SSI at the ID and midsection in c*Fgf13^KO^* cardiomyocytes (compared with WT cells) was due to the resulting perturbation of membrane cholesterol. Attempts to perform macropatch recordings in the presence of membrane cholesterol–modulating agents, such as MβCD or exogenous cholesterol, were unsuccessful. Cardiomyocytes displayed heightened sensitivity to these treatments, resulting in membrane instability, rapid contractions, and loss of seal formation during recordings. The use of a high-potassium bath solution, required to maintain the membrane potential near 0 mV, may have contributed to this instability, limiting the ability to obtain reliable recordings under these conditions. Since we observed that FGF13 affected SSI at either the ID or midsection, we recorded SSI in cardiomyocytes in the whole-cell configuration, which was amenable to cholesterol manipulation. Consistent with results in HEK293 cells, depletion of cholesterol with MβCD hyperpolarized the V_1/2_ of SSI recorded in WT or c*Fgf13^KO^* cardiomyocytes (Figure 8, A and B, and Supplemental Table 1). Addition of cholesterol to c*Fgf13^KO^* cardiomyocytes depolarized the V_1/2_ of SSI but did not affect the V_1/2_ of SSI in WT cardiomyocytes (Figure 8, C and D, and Supplemental Table 1). Again, results in HEK293 cells were analogous and together suggest that the effects of cholesterol on SSI in WT cardiomyocytes are saturated.

**Figure 9:**
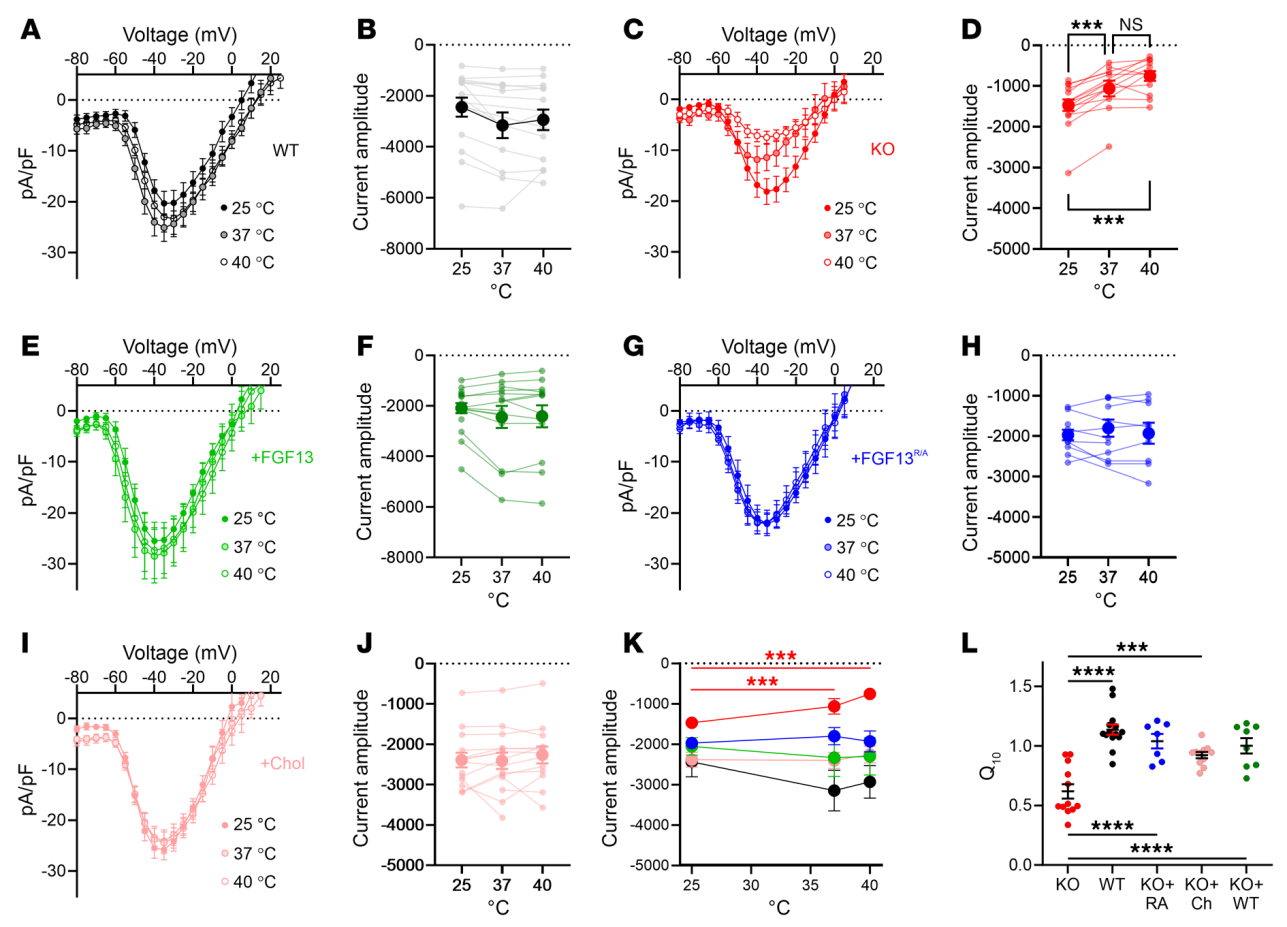
FGF13 and cholesterol protect VGSC currents at elevated temperatures in cardiomyocytes. (**A**, **C**, **E**, **G**, and **I**) Current-voltage relationships for sodium currents in WT (**A**), KO (**C**), KO + FGF13 (**E**), KO + FGF13^R/A^ (RA) (**G**), and KO + cholesterol-enriched (**I**) groups. (**B**, **D**, **F**, **H**, and **J**) Peak current changes in response to increased temperature in WT (**B**), KO (**D**), KO + WT FGF13 (**F**), KO + FGF13^R/A^ (**H**), and KO + cholesterol-enriched (**J**) groups. (**K**) Summary data of peak current density changes with temperature. (**L**) Calculated Q_10_ (ratio of the current per 10°C increase in temperature from 25°C) for the tested conditions. Statistical analysis: 1-way ANOVA with Bonferroni’s post hoc test (***P < 0.001, ****P < 0.0001).

Beyond affecting SSI, a previous report showed that *Fgf13* ablation in cardiomyocytes reduced VGSC currents at elevated temperatures, leading to a temperature-dependent conduction block (26); analogous data from neuron-specific *Fgf13*-KO models showed reduced heat nociception in dorsal root ganglion neurons (mediated by Na_V_1.7 channels) (55, 56). Since cholesterol affects membrane fluidity in a temperature-dependent fashion, we hypothesized that the observed conduction block was driven at least in part by the FGF13-dependent change in membrane cholesterol. To test our hypothesis, we first asked if the effects of temperature on Na_V_1.5 channels were independent of a direct interaction between FGF13 and Na_V_1.5 by performing paired recordings at 25°C and 40°C in HEK293 cells expressing Na_V_1.5 and FGF13 (or GFP as a control). We observed a marked reduction of Na_V_1.5 current amplitude at 40°C (Supplemental Figure 7, A and B, and Supplemental Table 1). In contrast, current amplitude at 40°C was not reduced when FGF13 or the binding-incompetent FGF13^R/A^ were coexpressed (Supplemental Figure 7, C–F, and Supplemental Table 1), showing that FGF13 preserves VGSC current amplitude at elevated temperatures independent of FGF13 binding to the channel. Similar to the protection afforded by coexpression of either FGF13 or FGF13^R/A^, we observed that addition of cholesterol to the membrane was also protective (Supplemental Figure 7, G and H, and Supplemental Table 1). The addition of FGF13, FGF13^R/A^, or cholesterol all increased the Q_10_ above that observed in cells expressing Na_V_1.5 and GFP only. We obtained congruent data in ventricular cardiomyocytes, observing no reduction in current amplitude when the bath temperature for WT cells increased from 25°C to 40°C (Figure 9, A and B, and Supplemental Table 1), but there was a significant reduction of current amplitude of c*Fgf13^KO^* cells in which the bath temperature was raised to 37°C or 40°C (Figure 9, C and D, and Supplemental Table 1). Moreover, viral expression of either WT FGF13 or the binding-incompetent FGF13^R/A^ in cardiomyocytes isolated form c*Fgf13^KO^* animals prevented the reduction in current amplitude at elevated temperatures (Figure 9, E–H, and Supplemental Table 1), demonstrating that the acute expression of FGF13 can provide temperature stability to VGSC currents in cardiomyocytes and that the effect of FGF13 is independent of Na_V_1.5 binding. Since cholesterol and temperature both affect membrane fluidity, we tested whether addition of cholesterol to c*Fgf13^KO^* cardiomyocytes stabilized VGSC currents as temperature increased. We added cholesterol to c*Fgf13^KO^* cells and then recorded VGSC current amplitudes while raising the bath temperature. Similar to viral expression of FGF13, addition of cholesterol prevented the reduction in current amplitude observed in c*Fgf13^KO^* cardiomyocytes (Figure 9, I and J, and Supplemental Table 1). Thus, like FGF13 (present in WT cardiomyocytes) or after viral expression of FGF13 or FGF13^R/A^ in c*Fgf13^KO^* cardiomyocytes, cholesterol protects VGSC current at elevated temperatures (Figure 9K) and increased the Q_10_ compared with the values obtained in c*Fgf13^KO^* cardiomyocytes (Figure 9L). These results therefore validate and extend the observations that FGF13-dependent effects on Na_V_1.5 SSI are both independent of Na_V_1.5 interaction and correlate with changes in local membrane cholesterol.

## Discussion

Our data redefine and expand the roles for members of the FHF subfamily of FGFs and offer insights into how channel auxiliary proteins affect the function of their regulatory targets, the channel pore–forming subunits. In cardiomyocytes, previous studies focused almost exclusively on how these FHFs serve as VGSC binding partners and regulate VGSC (8, 17, 19–22, 24–28, 32, 39, 45, 53, 57–60). This focused paradigm derived from a yeast 2-hybrid screen that identified FGF12 as an interactor with the cytoplasmic C-terminus of Na_V_1.9 (*SCN11A*) and was bolstered by studies that discovered variants in a neuronal FHF associated with ataxias and variants in cardiomyocyte FHFs associated with arrhythmias. Observations of perturbed VGSC function when channels were coexpressed with disease-associated variants provided pathogenic mechanisms to reinforce that paradigm (20, 22–24, 61, 62). Crystal structures showing the direct interaction between FHFs and the cytoplasmic C-termini of VGSCs (30, 31, 63, 64) provided additional support. Building upon the identification of FHFs as regulators of Na_V_1.5 trafficking and targeting in cardiomyocytes (19, 25), roles in the trafficking and targeting of other ion channels have also been proposed after the observation that FHFs affect currents beyond those of VGSCs (25, 65).

As FHFs are members of the FGF superfamily, however, VGSC regulation is not a priori an expected function, suggesting that FHFs have alternative roles. Indeed, because *FGF13* is expressed in neurons and was associated with intellectual disability (66), an investigation showed how FGF13 stabilizes microtubules in the brain, leading to the regulation of growth cones during neuronal development (14). Regulation of microtubules was subsequently described in cardiomyocytes (35, 36). Additional studies have implicated FGF13 in other functions that are independent of VGSCs, including control of neuronal excitability (65) and regulation of the number of caveolae in ventricular cardiomyocytes (45).

Those observations, along with confronting the paradox that the cytoplasmic FHFs affect VGSC SSI (the most commonly reported effect of FHFs on VGSCs, yet a process largely dependent on actions of membrane-delimited voltage sensors within the VGSCs), formed the impetus to investigate whether FGF13 affected VGSC function independent of binding. Here, we revealed a novel and unexpected membrane-dependent mechanism controlled by the cytoplasmic FGF13, specifically showing that FGF13 regulated changes in local membrane cholesterol, thereby affecting VGSC SSI. Manipulating membrane cholesterol content alters membrane stiffness, thereby influencing voltage-gated ion channel behavior (43, 44). Along with previous observations, our data here showing that *Fgf13*-KO affects local membrane cholesterol in cardiomyocytes, that addition or depletion of cholesterol affects VGSC SSI, and that addition of cholesterol can restore SSI V_1/2_ values in *Fgf13-*KO cardiomyocytes toward WT values suggest that at least some of the FHF effects on VSGC SSI are mediated by regulation of membrane cholesterol via a VGSC binding-independent mechanism. Cholesterol within the membrane can interact with proteins at cholesterol recognition/interaction amino acid consensus (CRAC) and inverted CRAC sequences (CARC) (67). Inspection of the cardiac VGSC Na_V_1.5 sequence identified a CRAC sequence and 3 CARC sequences within the channel’s transmembrane domains. The CRAC sequence and 1 of the CARC sequences are in D_III_S_2_ (Supplemental Figure 8A), and D_III_ has been implicated in regulating channel inactivation (68). We also examined a cryo-electron microscopy structure of the homologous Na_V_1.6 that was generated in complex with FGF13 (69); this showed cholesterol molecules adjacent to D_II_ and D_III_ (Supplemental Figure 8B). While there may be differences among specific VGSCs regarding cholesterol interaction, the consistent effects on channel inactivation for those channels studied (42–44, 47) and their sequence homology within their membrane-spanning segments suggest that cholesterol interactions and consequent effects are likely similar among the VGSC family.

In contrast to the voltage dependence of SSI, the kinetics of Na_V_1.5 fast inactivation depend upon direct interaction of FGF13 as the binding-incompetent FGF13^R/A^ cannot restore the time constant to WT levels when expressed in a c*Fgf13^KO^* cardiomyocyte. Thus, FGF13 appears to affect VGSC kinetics in cardiomyocytes via binding-dependent and -independent actions. Because the affinities of FHFs for the Na_V_1.5 cytoplasmic C-terminus are high (30, 59), we posit that an FHF bound to a Na_V_1.5 C-terminus is not a member of the pool that regulates local membrane cholesterol content and thereby affects SSI.

Not only do our data support and extend the previous observation (45) that FGF13 affects local membrane cholesterol, but our imaging data with ALOD4 demonstrate that FGF13 is responsible for a previously unreported concentration of accessible cholesterol at the IDs compared with other cardiomyocyte membrane areas. The mechanisms underlying this polarized distribution of accessible cholesterol in cardiomyocytes are not known. Nevertheless, this direct visualization of cholesterol concentrated at the ID is consistent with a previous study in which acute depletion with MβCD specifically disrupted ID integrity within intact hearts (70).

What are the roles of this FGF13-dependent concentration of accessible cholesterol at the ID? We postulate several consequences. First, FGF13 via increasing local accessible cholesterol at the ID supports increased Na_V_1.5 channel availability within the subcellular compartment where Na_V_1.5 channels are concentrated, thus promoting cardiomyocyte excitability and thereby increased conduction velocity. Indeed, in previous *Fgf13* knockdown (19) or KO (25, 26, 57) models, cardiac conduction was impaired. Here, we show that loss of FGF13-dependent regulation of local cholesterol at the ID may be another contributor to slowed conduction velocity. Second, we found that the previously reported protection by FGF13 of VGSCs in cardiomyocytes at elevated temperatures (26) is independent of FGF13 binding to Na_V_1.5 and that addition of cholesterol restored the protection to VGSC currents that was absent in c*Fgf13^KO^* cardiomyocytes. Intriguingly, the human *Fgf13* homolog, *FGF12*, was identified as a candidate locus for BrS (20), an arrhythmia exacerbated by febrile illnesses. Perhaps *FGF12* variants associated with BrS offer less protection to Na_V_1.5 currents at elevated temperatures.

Third, we observed that FGF13 knockout affected the concentration of Na_V_1.5 at the IDs (Figure 5, A–D), an effect previously attributed Na_V_1.5 interaction with SAP97 (71) and ankyrin-G (72). Our results suggest that FGF13 also contributes. Since cholesterol depletion with MβCD reduced Na_V_1.5 at the ID (Figure 5, E–G) suggests that the mechanism by which FGF13 promotes Na_V_1.5 concentration at the ID is also related to FGF13’s role in regulation of local membrane cholesterol.

The observation that the FGF13-dependent effects on local membrane cholesterol and on Na_V_1.5 SSI are independent of the interaction with Na_V_1.5 channels implies that the consequences of FGF13 on local membrane cholesterol are not limited to VGSCs. In a previous study, assessment of the effects of *Fgf13*-KO on ventricular cardiomyocyte action potentials suggested that FGF13 also regulates various cardiac Κ^+^ channels, yet there was no evidence that FGF13 interacted with specific K^+^ channels (25), consistent with our previous unbiased screen for FGF13 interactors by immunoprecipitation in which we did not identify K^+^ channels (45). Thus, the observed consequences on K^+^ channel currents in the *Fgf13*-KO model likely result from indirect FGF13 effects. As local membrane cholesterol is a well-described regulator of cardiomyocyte K^+^ channel currents (71, 72), these observations suggest that the mechanism by which FGF13 affects Na_V_1.5 channels may be more broadly applicable.

Finally, these results suggest that assessment of regulatory effects of ion channel auxiliary subunits should be investigated to determine if all effects depend upon direct binding between the auxiliary subunit and the pore-forming subunit. Many proteins that act as channel auxiliary subunits have identified roles beyond channel regulation. Only separation-of-function experiments, as we performed with FGF13^R/A^ here, can determine if observed effects on channel function depend upon the direct interaction between the auxiliary subunit and the pore-forming subunit or upon other functions of the auxiliary subunit independent of its binding to the channel pore.

## Methods

### Sex as a biological variable

Since *Fgf13* is an X-linked gene and complete *Fgf13-*KO is lethal (65, 73, 74), the c*Fgf13^KO^* mice studied here were males, obtained as described below.

### FGF13 constitutive KO animals

All mice were maintained on a C57BL/6J genetic background (000664; The Jackson Laboratory). To generate cardiac-specific constitutive KO mice, we crossed female *Fgf13^fl/fl^* (25) with hemizygous male *Myh6-Cre* (αMyh6-Cre; 011038; The Jackson Laboratory) mice, which have an α-myosin heavy chain promoter driving expression of Cre recombinase. Experiments were performed on 6- to 12-week-old mice.

### HEK cell transfection for electrophysiology

HEK293 cells were maintained in DMEM supplemented with 10% FBS and 1% penicillin-streptomycin under standard culture conditions (37°C, 5% CO_2_). For transfection, Lipofectamine 2000 (Thermo Fisher Scientific) was used according to the manufacturer’s protocol. Cells were transfected with 4 μg of Na_V_1.5 plasmid along with either 1 μg of GFP or 1 μg of FGF13-GFP or FGF13^R/A^-GFP plasmid. Transfected cells were maintained for 48 hours before being utilized for electrophysiological recordings or immunocytochemical analysis. Data generated from the same batch of cells, transfected simultaneously, were used to create graphs and perform statistical analyses to account for potential batch-to-batch variations.

### Immunoprecipitations of Na_V_1.5 with FGF13 and FGF13^R/A^

HEK293 cells were transfected with 2 μg of plasmid expressing Na_V_1.5 (30) and 0.4 μg of FGF13 (VY splice variant) WT or R120A mutant DNA with 6 μL of Lipofectamine 2000 and incubated for 24 hours at 37°C. Cells were washed 3 times with ice-cold PBS, harvested, and lysed in 0.3 mL of ice-cold lysis buffer (20 mM Tris-HCl, pH 8, 150 mM NaCl, 2 mM EDTA, and 1% IGEPAL CA-630 [MilliporeSigma]) supplemented with cOmplete Mini EDTA-free Protease Inhibitor Cocktail (Roche) and 0.2 mM PMSF. Lysates were centrifuged for 15 minutes at 17,000*g* and 4°C. Protein concentration was measured by Bradford assay (Pierce). For the immunoprecipitation, lysates were precleared with 20 μL of pre-equilibrated Protein G magnetic beads (Thermo Fisher Scientific) for 1 hour at 4°C. Fifty microliters was collected for Western blot analysis. Precleared lysates were incubated with 2 μL (~2 μg) of anti–V5-Tag monoclonal antibody (Thermo Fisher Scientific; R960-25) for 16 hours at 4°C. Immunoprecipitation was performed using 40 μL of pre-equilibrated Protein G magnetic beads for 1 hour at room temperature. Beads were washed 3 times in lysis buffer, and proteins were eluted in 80 μL of elution buffer (200 mM glycine and 50 mM Tris-HCl, pH 2.6) and immediately neutralized in 40 μL of 1 M Tris-HCl, pH 8.

Samples were analyzed by SDS-PAGE on an 8%–16% Tris-glycine gel transferred to a PVDF membrane using iBlot3 (Thermo Fisher Scientific). Membranes were blocked in blocking buffer (3% BSA [m/v] and 0.1% Tween 20 [v/v]) for 1 hour at room temperature and incubated overnight with primary antibodies diluted in blocking buffer at 4°C: rabbit anti-Na_V_1.5 (Alomone; 493-511) 1:1,000; custom rabbit anti-FGF13 antibody, previously described (19); and mouse anti-GAPDH (Thermo Fisher Scientific; MA5-15738) 1:1,000. Membranes were washed 5 times in TBST washing solution (150 mM NaCl, 20 mM Tris, pH 7.6, and 0.1% Tween 20 [v/v]) and incubated with secondary antibodies diluted in blocking buffer for 1 hour at room temperature: goat anti-rabbit IgG, HRP-linked (Cell Signaling Technology; 7074) 1:5,000 and anti-mouse m-IgGκ BP-HRP (Santa Cruz Biotechnology; sc-516102). Membranes were washed 5 times in TBST washing solution, incubated in SuperSignal West Pico PLUS substrate solution (Thermo Fisher Scientific), and imaged using a ChemiDoc Imager (Bio-Rad).

### Cardiomyocyte isolation, cell culture, and adenoviral transduction

Ventricular cardiomyocytes were isolated from left and right ventricles of *Myh6-Cre*^–^ (WT) and *cFgf13^KO^* mice using a previously validated Langendorff-free cell isolation method (75). Briefly, after anesthesia with 2,2,2-Tribromoethanol (Sigma Aldrich), the chest was incised to expose the heart and the inferior vena cava, and the descending aorta was cut. The heart was then flushed by injecting 10 mL of room temperature EDTA buffer (130 mM NaCl, 5 mM KCl, 0.5 mM NaH_2_PO_4_, 10 mM HEPES, 10 mM glucose, 10 mM 2,3-butanedione 2-monoxime [BDM], 10 mM taurine, and 5 mM EDTA) in the right ventricle. After placing an aortic clamp, the heart was excised and transferred to a 60 mm dish containing EDTA buffer. Next, 10 mL of room temperature EDTA buffer was injected into the apex of the left ventricle, and the same aperture was used to inject 5 mL of perfusion buffer (130 mM NaCl, 5 mM KCl, 0.5 mM NaH_2_PO_4_, 10 mM HEPES, 10 mM glucose, 10 mM BDM, 10 mM taurine, and 1 mM MgCl_2_, warmed to 37°C). After injection of perfusion butter, collagenase buffer (0.5 mg/mL collagenase II, 0.5 mg/mL collagenase IV, and 0.05 mg/mL protease XIV) was serially injected (5 × 10 mL) into the left ventricle. The atria were separated and discarded. The ventricles were pulled into approximately 1 mm pieces with forceps, and cells were dissociated with gentle titration for 2 minutes. Enzymatic activity was inhibited by addition of 3 mL of stop buffer (perfusion buffer with 5% sterile FBS, made fresh), and the cell suspension was passed through a 150 mm filter before cells were allowed to settle by gravity for 20 minutes. The cells were then washed twice in perfusion buffer and allowed to settle by gravity for 10 minutes each before use in various experiments.

Cell culture and adenoviral infection of mouse ventricular myocytes were performed as described (19, 75). Briefly, isolated cardiomyocytes were placed in precoated wells with laminin (5 μg/mL in PBS for 1 hour at 37°C before laminin was aspirated and well washed with PBS) in 12-well plates using plating media (made in M199 [Thermo Fisher Scientific] supplemented with 5% FBS, 10 mM BDM, and 1× penicillin/streptomycin). Cells were allowed to adhere for 1 hour before plating media was removed and replaced with 500 μL of culture media (made in M199 supplemented with 0.1% BSA, 10 mM BDM, 1× chemically defined lipid concentrate [Thermo Fisher Scientific], 1× insulin-transferrin-selenium [Sigma-Aldrich], and penicillin-streptomycin). After 2 hours in culture media, a subset of *Fgf13^KO^* cardiomyocyte wells was infected with 1.5 μL of Cre-dependent AAV8-DIO-FGF13 virus (adeno-associated virus serotype 8, double-floxed inverted orientation), which encodes the FGF13-VY splice variant (65) and is the most highly expressed variant in mouse heart (19) (1.6 × 10^8^ infectious units [ifu]/mL). Another subset of KO cardiomyocyte wells was infected with 1.5 μL of AAV8-DIO-FGF13^R/A^ (6.6 × 10^7^ ifu/mL). Cardiomyocytes were incubated in culture media for 48 hours for adequate viral expression before subsequent analyses.

### Electrophysiology

All experiments were conducted at room temperature unless otherwise specified. Whole-cell voltage-clamp recordings were performed using EPC10 amplifier with Patchmaster software for data acquisition and Fitmaster software for analysis (HEKA Elektronik). Sodium channel recordings shown in Figures 1 and 2, as well as recordings in HEK293 cells, were performed with an external solution containing (in mM) 124 NaCl, 5 KCl, 2 CaCl_2_, 1 MgCl_2_, 20 TEA-Cl, 5 HEPES, and 10 glucose, adjusted to pH 7.4 with NaOH. Borosilicate glass pipettes were filled with an internal solution containing (in mM) 125 CsF, 10 NaCl, 10 HEPES, 15 TEA-Cl, 1.1 EGTA, and 0.5 Na-GTP, adjusted to pH 7.3 with NaOH (8). Pipettes were prepared using a P-97 pipette puller (Sutter Instruments) and had a resistance of 0.8–1.2 MΩ.

For sodium channel recordings presented in the remaining figures, cells were perfused with an external solution containing (in mM) 7.0 NaCl, 133.0 CsCl, 1.8 CaCl_2_, 1.2 MgCl_2_, 11.0 glucose, 5.0 HEPES, and 0.005 nifedipine, adjusted to pH 7.4 with CsOH. The internal pipette solution contained (in mM) 3.0 NaCl, 133.0 CsCl, 2.0 MgCl_2_, 2.0 Na_2_ATP, 2.0 TEA-Cl, 10.0 EGTA, and 5.0 HEPES, adjusted to pH 7.3 with CsOH (76). Pipettes had a resistance of 2.5–3.0 MΩ. All the components used for external and internal solutions were obtained from Sigma-Aldrich.

Macropatch recordings were conducted in the cell-attached configuration as previously described (76) to measure Na_V_1.5 currents from the IDs and lateral membranes of isolated cardiac myocytes. Recording pipettes (resistance 0.8–1 MΩ) were prepared as described previously. The pipette solution contained (in mM) 140 NaCl, 5.4 KCl, 1.8 CaCl_2_, 1.0 MgCl_2_, 5.5 glucose, 5.0 HEPES, and 0.005 nifedipine, adjusted to pH 7.4 with NaOH. The bath solution consisted of (in mM) 145.2 KCl, 1.8 CaCl_2_, 1.0 MgCl_2_, 5.5 glucose, and 5.0 HEPES, adjusted to pH 7.4 with KOH. This ionic configuration maintained the membrane potential close to 0 mV. All the components used for external and internal solutions were obtained from Sigma-Aldrich.

For current-voltage curves, cells were held at –120 mV and depolarized to a series of test voltages ranging from –90 to +55 mV. To generate SSI curves, currents were elicited at –20 mV for 20 ms following a 500-ms prepulse to test voltages ranging from –120 to +20 mV from a holding potential of –120 mV. Normalized currents at the –20 mV pulse were used to construct the SSI curve. The data were fitted using a Boltzmann equation, I/Imax = 1/[1 + exp(V − V_1/2)_/k)], where Imax is the maximum current, V_1/2_ is the half-inactivation voltage, and k is the slope factor. Activation curves were calculated by computing conductance (G, where G = I[(V – Vrev)]) from the measured current using a reversal potential (Vrev) calculated for the specific solutions used (see above). The conductance values were then fitted to a Boltzmann equation, G/Gmax = 1/[1 + exp(V − V_1/2)_/k)], where Gmax is the maximum conductance, V_1/2_ is the half-activation voltage, and k is the slope factor.

To calculate the time constant (τ) of current decay during sodium channel inactivation, the decay phase of the sodium current was fitted to a single-exponential function, I(*t*) = A × exp(− *t*/τ) + A0, where I(*t*) is the total current at time *t*, A is the amplitude of the decaying component of the current at *t* = 0, τ is the time constant of current decay, and A0 is the nondecaying steady-state current. Analyses were performed at different test voltages to determine the voltage dependence of the decay kinetics.

For temperature-dependent experiments, the HEK cells and myocytes were initially patched at room temperature (25°C). After recording the sodium channel currents at room temperature, the temperature of the perfusing bath solution was gradually increased to approximately 37°C and/or 40°C and the sodium channel currents were recorded at each temperature level.

### Cell imaging

#### Immunocytochemistry with antibodies.

Acutely isolated or cultured cardiac myocytes were fixed with 2% paraformaldehyde for 15 minutes at room temperature. Cells were then permeabilized with 0.2% Triton X-100 in antibody diluent solution for 15 minutes, followed by blocking in antibody diluent solution for 1 hour. Subsequently, cells were incubated with the primary antibody overnight at 4°C. After washing 3 times with PBS, cells were incubated with the secondary antibody for 1 hour at room temperature. Following another 3 washes with PBS, coverslips containing the stained cells were mounted on slides using mounting medium and allowed to dry before imaging. Antibodies used were rabbit anti-Na_V_1.5 (Alomone; 493-511; 1:500) and mouse anti–N-Cad (Santa Cruz Biotechnology; sc-59987; 1:500).

#### Staining with ALOD4.

For experiments involving ALOD4 staining, acutely isolated myocytes or HEK293 cells were preincubated with 3 μM ALOD4 (provided by Arun Radhakrishnan, UT Southwestern Medical Center, Dallas, Texas, USA) conjugated to Alexa Fluor 568 for 20 minutes at 37°C in culture. After incubation, cells were washed 3 times with PBS and subsequently fixed with 2% paraformaldehyde for 15 minutes. Following fixation, cells were washed 3 more times with PBS and stained with wheat germ agglutinin for 45 minutes at room temperature. Cells were then washed again 3 times with PBS, mounted on slides using mounting medium, and allowed to dry prior to imaging.

### Preparation and application of cholesterol and MβCD solutions

Cholesterol and MβCD were purchased from Sigma-Aldrich and prepared as previously described (43). Culture media used for HEK cells and myocytes served as the diluent. MβCD was prepared by dissolving the appropriate amount of powdered MβCD in the media. Cholesterol was then added to the solution at a 1:10 molar ratio (cholesterol/MβCD). The mixture was sonicated for 10 minutes and incubated with continuous rotation in a tube overnight at 37°C. The following day, the solution was filtered through a 0.22 μm filter prior to application to the cells. Cells were treated with either 500 μM MβCD solution for 1 hour to deplete cholesterol or 500 μM MβCD + cholesterol solution to enrich cholesterol.

#### In-gel imaging for ALOD4.

HEK293 cells were transfected with 1 μg of eGFP or FGF13VY-eGFP WT or R120A mutant DNA with 3 μL of Lipofectamine 2000 and incubated for 36 hours at 37°C. Cells were incubated at 37°C with cholesterol or MβCD for 1 hour, then with ALOD4 as described above. Cells were washed 3 times in ice-cold PBS, harvested, and lysed in ice-cold RIPA buffer (50 mM Tris-HCl, 150 mM NaCl, 0.1% Triton X-100 [v/v], 0.5% sodium deoxycholate [v/v], and 0.1% SDS) supplemented with cOmplete Mini EDTA-free Protease Inhibitor Cocktail and 0.2 mM PMSF. Lysates were centrifuged for 15 minutes at 17,000*g* and 4°C. Protein concentration was measured by Bradford assay. Samples were analyzed by SDS-PAGE on an 8%–16% Tris-glycine gel. ALOD4 was imaged in-gel using the Alexa Fluor 647 filter of the ChemiDoc Imager (Bio-Rad).

#### Staining with filipin.

For filipin staining, acutely isolated myocytes were fixed with 2% paraformaldehyde for 15 minutes at room temperature. Cells were washed 3 times with PBS and incubated with 50 μg/mL filipin solution prepared in PBS for 45 minutes at room temperature. After staining, cells were washed 3 additional times with PBS and mounted on slides using mounting medium.

#### Imaging.

Images of antibody and ALOD4 staining were acquired using a Zeiss LSM 800 confocal microscope. Filipin-stained cells were imaged on a Leica Thunder microscope, with excitation at 405 nm.

#### Image analysis.

Line scan analysis was performed to quantify the relative intensity distribution along the myocyte membrane. Using the Profile feature in Zeiss Zen 3.10 Lite software, a 25-pixel-wide line was drawn starting 1 μm before the ID and extending into the lateral membrane of the cell. Intensity values along the line were quantified for all cells across experimental groups.

For filipin staining, the mean gray value for each cell was calculated across experimental groups using ImageJ software. These values were used to assess and compare fluorescence intensity levels.

### Statistics

Results are presented as mean ± SEM; the statistical significance of differences between groups was assessed using either a 2-tailed Student’s *t* test or 1-way ANOVA and was set at *P* < 0.05. Other statistical analyses were performed as previously described (45).

### Study approval

This study was approved by the Weill Cornell Institutional Animal Care and Use Committee (protocol 2016-0042), and all animals were handled in accordance with the NIH Guide for the Care and Use of Laboratory Animals. All mice were maintained on a C57BL/6J genetic background (000664; The Jackson Laboratory).

### Data availability

Data for all values reported in this article are available in the Supporting Data Values file.

## Author contributions

ARG, FRM, SOM, and GSP designed experiments. ARG, M Malvezzi, LTD, and M Matsui, performed experiments. ARG, M Malvezzi, KM, and GSP analyzed data. CIJM supplied reagents and performed the Alexa Fluor conjugation. ARG, SOM, and GSP wrote and edited the manuscript. 

## Funding support

This work is the result of NIH funding, in whole or in part, and is subject to the NIH Public Access Policy. Through acceptance of this federal funding, the NIH has been given a right to make the work publicly available in PubMed Central.

• NIH grants, R01 HL146149 and R01 HL160089, to GSP and SOM.

• American Heart Association Predoctoral Award, 25PRE1374923, to LTD.

• Medical Scientist Training Program grant, National Institute of General Medical Sciences, NIH T32GM152349, to the Weill Cornell/Rockefeller/Sloan Kettering Tri-Institutional MD-PhD Program (support of LTD).

## Supplementary Material

Supplemental data

Unedited blot and gel images

Supplemental table 2

Supporting data values

## Version 1 08/12/2025

In-Press Preview

## Version 2 10/15/2025

Electronic publication
