# Supplementary material for: The NaV1.5 auxiliary subunit FGF13 modulates channels by regulating membrane cholesterol independent of channel binding
Source: J Clin Invest. 2025 Aug 12;135(20):e191773. doi: 10.1172/JCI191773 (PMC12520690; doi:10.1172/JCI191773)
Supplement: Supplemental data [file jci-135-191773-s099.pdf]

**SUPPLEMENTAL DATA FOR:**

# The $Na_V1.5$ auxiliary subunit FGF13 modulates channels by regulating membrane cholesterol independent of channel binding

Aravind Gade[1], Mattia Malvezzi[1], Lala Tanmoy Das[1,2], Maiko Matsui[1], Cheng-I J. Ma[3], Keon Mazdisnian[1], Steven O. Marx[4], Frederick R. Maxfield[3], and Geoffrey S. Pitt[1,*]

**Supplemental Table 1:** Summary Electrophysiology data.

**Current density**

| | pA/pF | n |
|---|---|---|
| *Acutely isolated cardiomyocytes* | | |
| WT | -645.1 ± 57.3 | 20 |
| KO | -849.5 ± 134.9 | 12 |
| | | |
| *Cultured cardiomyocytes* | | |
| WT | -603.3 ± 81.8 | 17 |
| KO | -633.8 ± 56.1 | 24 |
| KO+FGF13 | -688.6 ± 49.4 | 30 |
| KO+FGF13$^{R/A}$ | -924.5 ± 84.2* | 18 |
| *, *P* <0.05 v. KO | | |
| | | |
| *Acutely isolated cardiomyocytes - macropatch* | | |
| WT - Mid | -92.1 ± 7.8 | 37 |
| KO - Mid | -131.1 ± 52.3 | 13 |
| WT - ID | -260.7 ± 42.0 **** | 13 |
| KO - ID | -117.6 ± 11.3 | 9 |

****, *P*<0.001 v WT - LM

**Tau (inactivation) @ -30 mV**

| | Tau (s) | n |
|---|---|---|
| *Acutely isolated cardiomyocytes* | | |
| WT | 1.79 ± 0.10 | 18 |
| KO | 1.08 ± 0.08 **** | 15 |

****, *P*<0.0001 v WT

| | Tau (s) | n |
|---|---|---|
| *Cultured cardiomyocytes* | | |
| WT | 2.15 ± 0.20 | 17 |
| KO | 1.00 ± 0.04 **** | 19 |
| KO+FGF13 | 2.19 ± 0.20 | 16 |
| KO+FGF13$^{R/A}$ | 1.49 ± 0.13 * | 12 |

*, *P*<0.0001 v WT; ****, *P*<0.0001 v WT

| | Tau (s) | n |
|---|---|---|
| *HEK293 Cells* | | |
| GFP | 1.55 ± 0.10 | 19 |
| FGF13 | 3.16 ± 0.40 ** | 9 |
| FGF13$^{R/A}$ | 1.76 ± 0.19 | 12 |

**, *P* <0.01 v GFP

**Peak current (temperature)**

| | pA | n |
|---|---|---|
| *Acutely isolated cardiomyocytes* | | |
| WT (25 °C) | -2444.0 ± 372.8 | 16 |
| WT (37 °C) | -3156.3 ± 503.6 | 12 |
| WT (40 °C) | -2939.9 ± 403.5 | 14 |
| | | |
| *Acutely isolated cardiomyocytes* | | |
| KO (25 °C) | -1473.2 ± 143.3 | 15 |
| KO (37 °C) | -1063.6 ± 191.6 *** | 10 |
| KO (40 °C) | -755.7 ± 120.4 *** | 12 |

***, *P* <0.001 v 25 °C

| | pA | n |
|---|---|---|
| *Acutely isolated cardiomyocytes* | | |
| KO+FGF13 (25 °C) | -2059.5 ± 198.8 | 19 |
| KO+FGF13 (37 °C) | -2340.6 ± 413.3 | 14 |
| KO+FGF13 (40 °C) | -2305.6 ± 444.7 | 12 |
| | | |
| *Acutely isolated cardiomyocytes* | | |
| KO+FGF13$^{R/A}$ (25 °C) | -1968.2 ± 130.0 | 11 |
| KO+FGF13$^{R/A}$ (37 °C) | -1797.0 ± 212.5 | 10 |
| KO+FGF13$^{R/A}$ (40 °C) | -1925.9 ± 261.2 | 9 |
| | | |
| *Acutely isolated cardiomyocytes* | | |
| KO+Chol (25 °C) | -2390.3 ± 182.7 | 14 |
| KO+Chol (37 °C) | -2407.9 ± 200.7 | 15 |
| KO+Chol (40 °C) | -2261.5 ± 211.4 | 13 |
| | | |
| *HEK293 Cells* | | |
| GFP (25 °C) | -2560.5 ± 251.7 | 18 |
| GFP (40 °C) | -2020.1 ± 271.9 *** | 18 |

***, *P* <0.001 v 25 °C

| | pA | n |
|---|---|---|
| *HEK293 Cells* | | |
| FGF13 (25 °C) | -1426.7 ± 409.3 | 10 |
| FGF13 (40 °C) | -1642.4 ± 561.9 | 10 |
| | | |
| *HEK293 Cells* | | |
| FGF13$^{R/A}$ (25 °C) | -2896.0 ± 820.4 | 6 |
| FGF13$^{R/A}$ (40 °C) | -3154.7 ± 920.1 | 6 |
| | | |
| *HEK293 Cells* | | |
| Chol (25 °C) | -3278.6 ± 343.3 | 7 |
| Chol (40 °C) | -3532.9 ± 313.2 | 7 |

**Steady state inactivation**

| | $V_{1/2}$ | | n |
|---|---|---|---|
| *Acutely isolated cardiomyocytes* | | | |
| WT | -82.1 ± | 1.0 | 34 |
| KO | -89.5 ± | 1.0 **** | 39 |

*, *P*<0.0001 v WT

| | | | |
|---|---|---|---|
| *Cultured cardiomyocytes* | | | |
| WT | -88.9 ± | 6.4 * | 14 |
| KO | -95.4 ± | 8.4 | 39 |
| KO+FGF13 | -85.0 ± | 7.6 **** | 37 |
| KO+RA | -87.1 ± | 9.2 *** | 31 |

*, *P*<0.05 v KO; ***, *P* <0.001 v KO; ****, *P*<0.0001 v KO

| | | | |
|---|---|---|---|
| *Cultured cardiomyocytes* | | | |
| WT+Chol | -0.8 ± | 1.2 | 24 |
| KO+Chol | 5.0 ± | 1.9 † | 17 |
| WT+MβCD† | -9.9 ± | 1.9 ++++ | 15 |
| KO+MβCD† | -21.8 ± | 1.5 †† | 9 |

Calculated as change in $V_{1/2}$ ($\Delta V_{1/2}$): †, *P*<0.05 v KO; ††, *P* <0.01 v KO; ++++ <0.0001 v WT

| | | | |
|---|---|---|---|
| *HEK293 Cells* | | | |
| GFP | -86.9 ± | 1.3 | 32 |
| FGF13 | -79.4 ± | 2.1 ** | 16 |
| $FGF13^{R/A}$ | -79.3 ± | 1.3 *** | 25 |

**, *P*<0.01 v GFP; ***, *P* <0.001 v GFP

| | | | |
|---|---|---|---|
| *HEK293 Cells* | | | |
| GFP+Chol | 8.2 ± | 1.9 ** | 15 |
| FGF13+Chol | 11.8 ± | 1.5 + | 6 |
| GFP+MβCD | -9.2 ± | 1.5 **** | 21 |
| FGF13+MβCD | 4.0 ± | 1.6 ++ | 20 |

Calculated as change in $V_{1/2}$ ($\Delta V_{1/2}$): **, *P*<0.01 v GFP; ****, *P*<0.001 v GFP; +, *P*=ns v FGF13; *P*<0.05 v FGF13

| | | | |
|---|---|---|---|
| *Acutely isolated cardiomyocytes - macropatch* | | | |
| WT - Mid | -96.6 ± | 1.0 * | 28 |
| KO - Mid | -102.3 ± | 1.8 ‡ | 13 |
| WT - ID | -91.8 ± | 1.5 | 13 |
| KO - ID | -99.3 ± | 1.2 * | 8 |

*, *P*<0.05 v WT - ID; ‡, *P*<0.05 v WT - Mid

**Activation**

| | $V_{1/2}$ | | n |
|---|---|---|---|
| *HEK293 Cells* | | | |
| GFP | -39.4± | 1.8 *** | 11 |
| GFP+MβCD | -48.8 ± | 1.7 | 12 |
| FGF13 | -38.5 ± | 1.5 | 6 |
| FGF13+MβCD | -39.4 ± | 1.3 | 20 |

***, *P*<0.01 v GFP+MβCD

**Supplemental Table 2:** Mass spectrometry data for proteins immunoprecipitated with $Na_V1.5$ from WT and c*Fgf13*$^{KO}$ hearts (sheet 1); Gene Ontology analyses (sheets 2 and 3).

**FHFs:**

```
sp|Q92914|FGF11         RPDRGPEPQLKGIVTKLFCRQGFYLQANPDGSIQGTPEDTSSFTHFNLIPVGLRVVTIQS  118
sp|P61328|FGF12         PVRRRPEPQLKGIVTRLFSQQGYFLQMHPDGTIDGTKDENSDYTLFNLIPVGLRVVAIQG  120
sp|Q92913|FGF13         R-RRRPEPQLKGIVTKLYSRQGYHLQLQADGTIDGTKDEDSTYTLFNLIPVGLRVVAIQG  126
sp|Q92915|FGF14         RL-RRQDPQLKGIVTRLYCRQGYYLQMHPDGALDGTKDDSTNSTLFNLIPVGLRVVAIQG  118
                           *  :********:*:.:**:.** : **:::** :: :  * ***********:**.
```

**Sodium channels:**

```
sp|P35498|SCN1A         NKLQLIAMDLPMVSGDRIHCLDILFAFTKRVLGESGEMDALRIQMEERFMASNPSKVSYQ  1904
sp|Q99250|SCN2A         NKVQLIAMDLPMVSGDRIHCLDILFAFTKRVLGESGEMDALRIQMEERFMASNPSKVSYE  1894
sp|Q9NY46|SCN3A         NKVQLIAMDLPMVSGDRIHCLDILFAFTKRVLGESGEMDALRIQMEDRFMASNPSKVSYE  1889
sp|P35499|SCN4A         NKIKLITLDLPMVPGDKIHCLDILFALTKEVLGDSGEMDALKQTMEEKFMAANPSKVSYE  1716
sp|Q14524|SCN5A         NQISLINMDLPMVSGDRIHCMDILFAFTKRVLGESGEMDALKIQMEEKFMAANPSKISYE  1890
sp|Q9UQD0|SCN8A         NTIELIAMDLPMVSGDRIHCLDILFAFTKRVLGDSGELDILRQQMEERFVASNPSKVSYE  1884
sp|Q15858|SCN9A         NKVQLIAMDLPMVSGDRIHCLDILFAFTKRVLGESGEMDSLRSQMEERFMSANPSKVSYE  1878
sp|Q9Y5Y9|SCN10A        NRNILIQMDLPLVPGDKIHCLDILFAFTKNVLGESGELDSLKANMEEKFMATNLSKSSYE  1840
sp|Q9UI33|SCN11A        NKYQFLVMDLPMVSEDRLHCMDILFAFTARVLGGSDGLDSMKAMMEEKFMEANPLKKLYE  1722
                        *   :: :***:*  *::**:*****:* .*** *. :* ::  **::*: :*  *  *:
```

**Figure S1: The binding sites in FHFs and VGSCs are conserved.** Sequence lineups of FHFs (FGF11-FGF14) with the critical FHF Arg and the critical VGSC His highlighted in red.

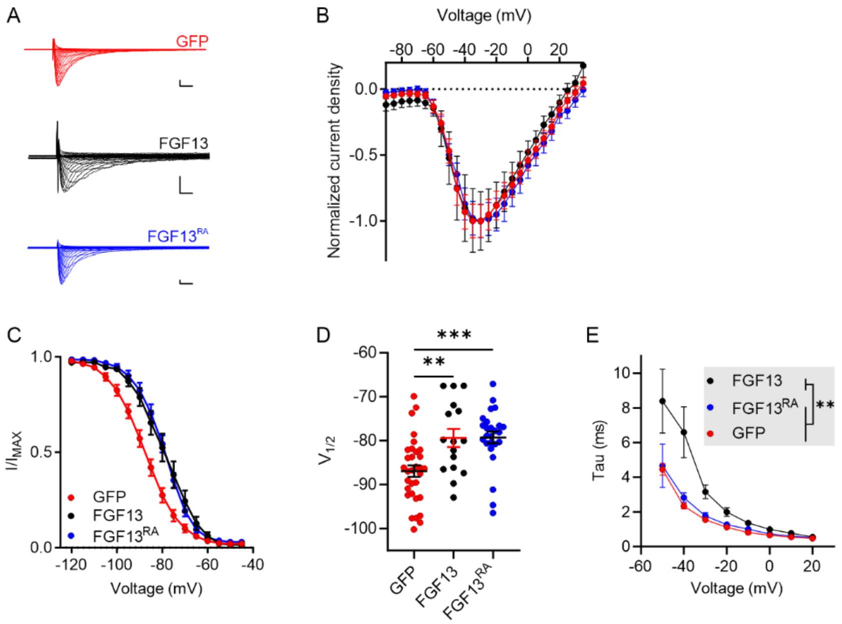


**Figure S2: A binding incompetent FGF13 mutant confers a subset of WT-like regulatory functions in HEK293 cells.** (A) Representative sodium current traces recorded from HEK cells co-expressing Nav1.5 with GFP, FGF13-GFP, or FGF13$^{R/A}$-GFP. Scale bars, 500 pA and 2 ms. (B) Normalized current-voltage relationships for the groups shown in (A). (C) Steady-state inactivation curves demonstrating normalized currents at -20 mV from different holding potentials. (D) V1/2 of inactivation values from (C), showing significant shifts in FGF13 and FGF13$^{R/A}$-expressing groups. Statistical analysis: One-way ANOVA with Bonferroni post-hoc tests (**p < 0.01, ***p < 0.001). (E) Time constants (Tau) of inactivation at various voltages, indicating effects of FGF13 but not FGF13$^{R/A}$. Statistical analysis: One-way ANOVA with Bonferroni post-hoc tests (**p < 0.01, ****p < 0.0001). (H) Normalized current-voltage relationships illustrating differences between FGF13 and FGF13$^{R/A}$ groups.

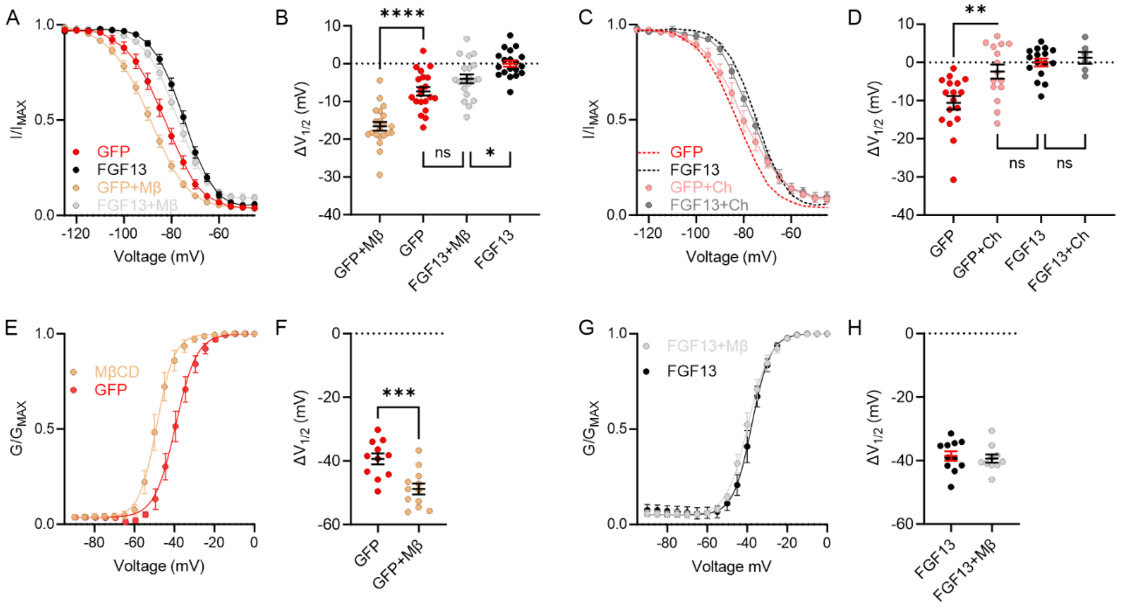


**Figure S3: FGF13 differentially affects consequences of manipulation of membrane accessible cholesterol affects SSI of $Na_V1.5$ in HEK293 cells.** (A, C) Steady-state inactivation curves of $Na^+$ currents in HEK cells expressing GFP or WT FGF13, treated with MβCD (cholesterol depletion) or cholesterol enrichment, respectively. (B, D) Change in the $V_{1/2}$ of inactivation values showing MβCD- and cholesterol-induced effects in GFP and FGF13 groups. Statistical analysis: One-way ANOVA with Bonferroni post-hoc tests (*p < 0.05, **p < 0.01, ****p < 0.0001). (E, G) Activation curves of $Na^+$ currents in HEK cells expressing GFP of WT FGF13, treated with MβCD. (F, H) Change in the $V_{1/2}$ of activation values showing MβCD-induced effects in GFP and FGF13 groups. Statistical analysis: Unpaired *t*-tests with Welch's correction (***p < 0.001).

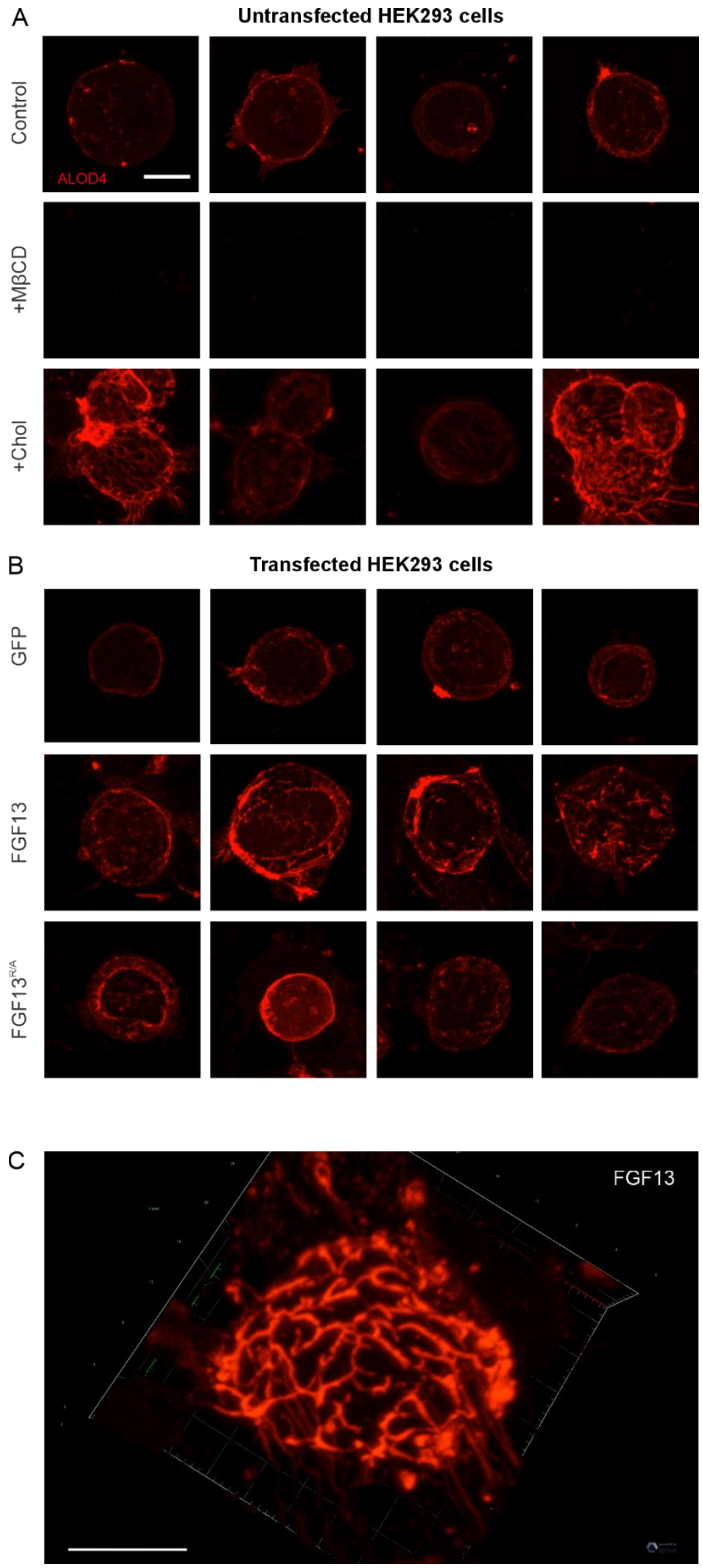


**Figure S4: Additional confocal images for Figure 3.** (A) Confocal images showing ALOD4 staining in HEK cells under control conditions and after MβCD treatment or cholesterol enrichment (Scalebar – 10 µm). (B) ALOD4 staining in HEK cells transfected with GFP, FGF13, or FGF13$^{R/A}$. (C) Three dimensional projection of a HEK cell transfected with FGF13 and imaged with ALOD4 (Scalebar – 10 µm).

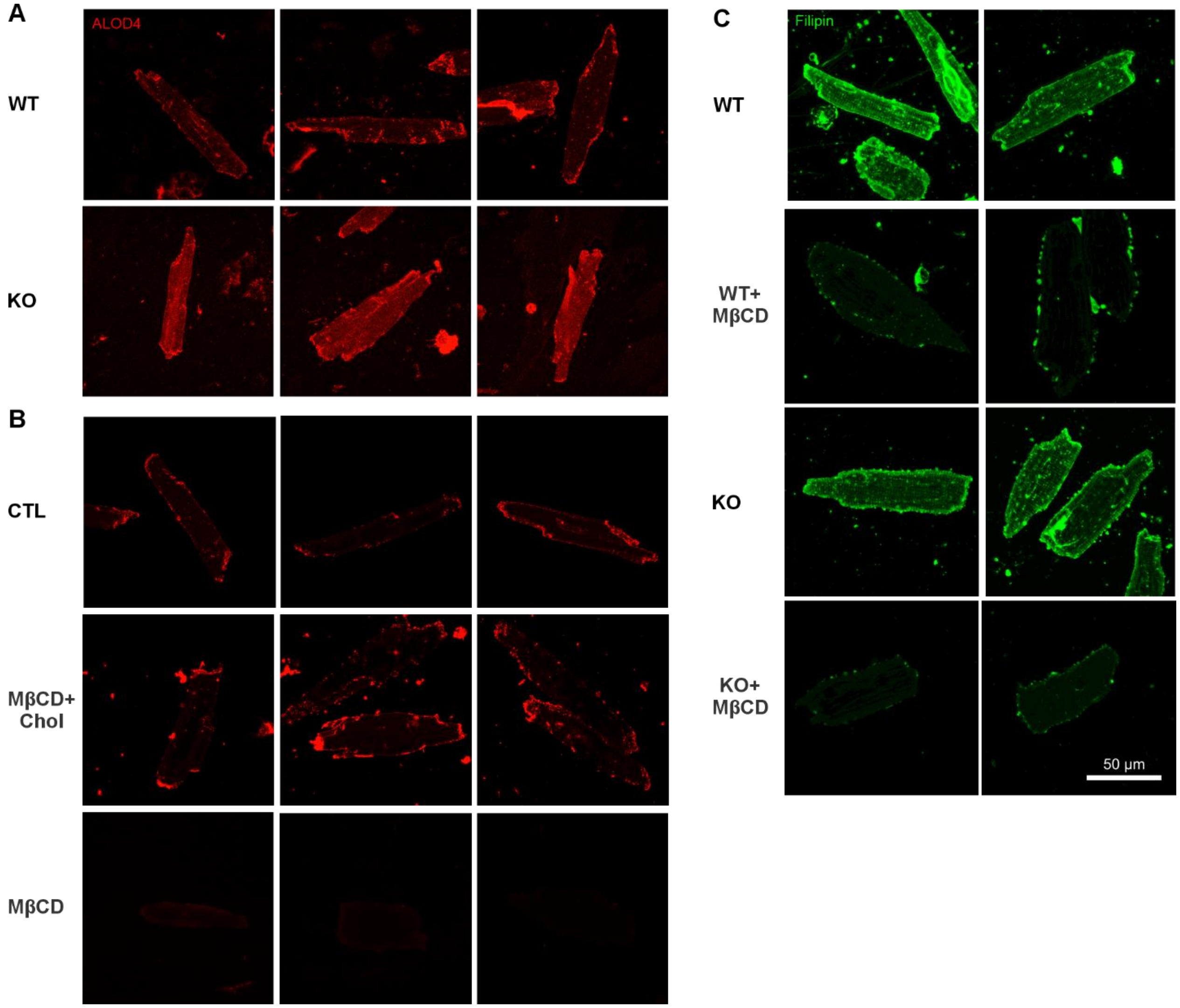


**Figure S5: Additional confocal images for Figure 4.** (A) Confocal images of ALOD4 staining in cardiac myocytes from WT and c*Fgf13*$^{KO}$ mice. (B) ALOD4 staining in WT myocytes under control conditions and after MβCD treatment or cholesterol enrichment. (C) Filipin staining of myocytes from WT and KO mice, under control and MβCD conditions (Scalebar – 50 µm).

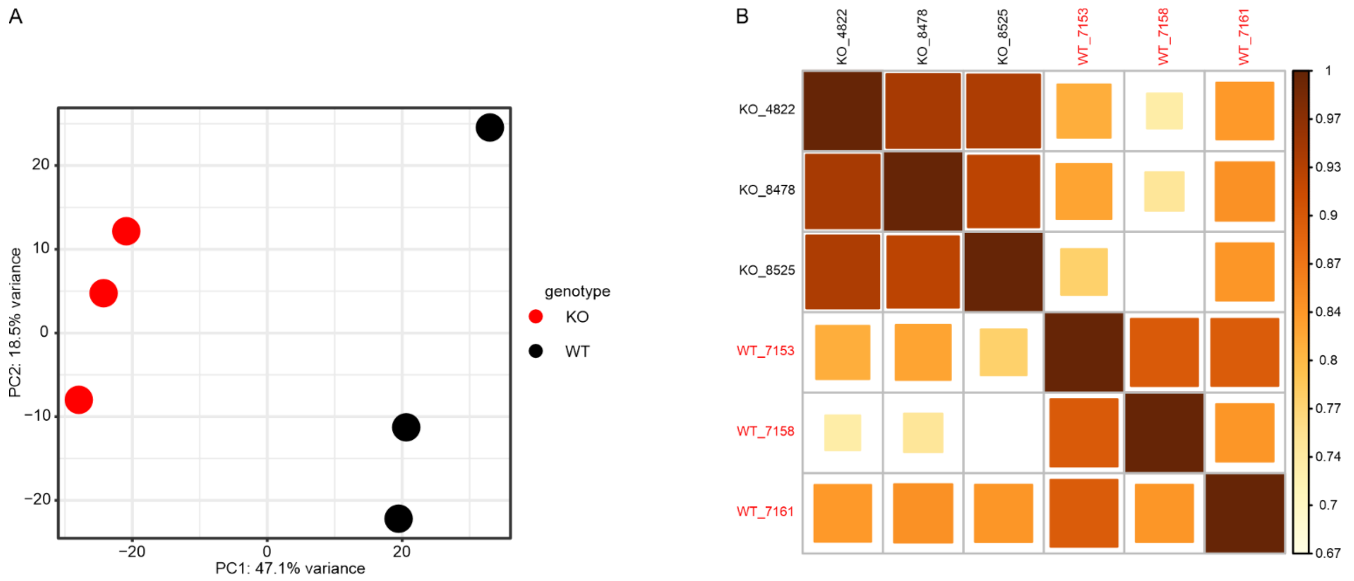


**Figure S6: Characterization of $Na_V1.5$ co-immunoprecipitation datasets from WT and c*Fgf13*$^{KO}$ hearts.** (A) Principal component analysis and (B) Pearson's correlations for the datasets from 3 WT and 3 c*Fgf13*$^{KO}$ hearts.

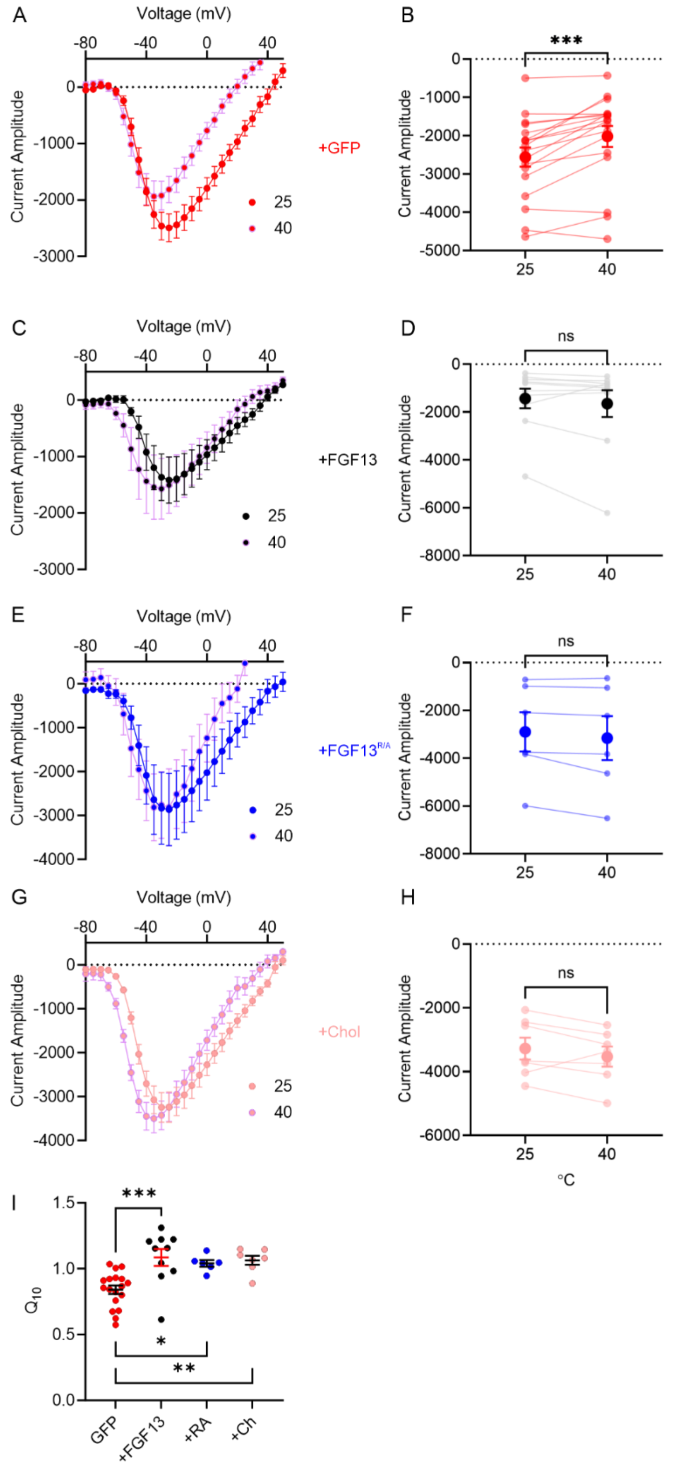


**Figure S7: FGF13 and cholesterol protect $Na_V1.5$ currents at elevated temperatures in HEK293 cells.** (A, C, E, G) Current-voltage relationships of sodium currents in HEK cells expressing GFP, FGF13, and $FGF13^{R/A}$, respectively at 25 and 40 °C. (B, D, F, H) Paired peak current amplitudes in response to increased temperature for the same conditions. Statistical analysis: Paired t-test (***p < 0.001). (I) Calculated $Q_{10}$ for the conditions tested. Statistical analysis: ANOVA with Dunnett's multiple comparisons tests (*p < 0.05, (**p < 0.01, ***p < 0.001).

A

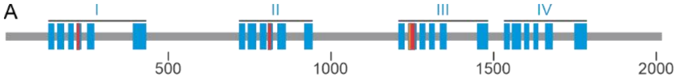


| CRAC | | CARC | |
|---|---|---|---|
| IIIS2 | 1239-VLLEYADK-1246 | IS4 | 219-RTFRVLRAL-227 |
| | | IIS4 | 808-RSFRLLRV-815 |
| | | IIIS2 | 1246-KMFTYVFVLEMLL-1258 |

B

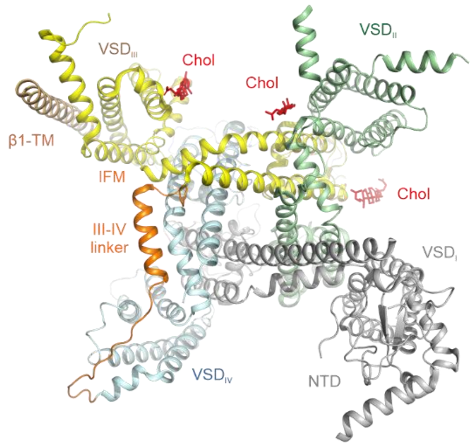


**Figure S8: Candidate cholesterol binding sites in Na$_V$1.5.** (A) Linear diagram of Na$_V$1.5 (numbers indicate amino acids) indicating the transmembrane segments (blue rectangles) and the domains (I-IV). Red or orange rectangles within the transmembrane segments indicate CRAC and CARC sequences, respective, which are indicated in the Table. (B) Na$_V$1.6 structure (PDB: 8FHD) with viewpoint from outside the cell looking into the channel's pore. The cholesterol or cholesterol hemisuccinate molecules are indicated.
