# Supplementary material for: The NaV1.5 auxiliary subunit FGF13 modulates channels by regulating membrane cholesterol independent of channel binding
Source: J Clin Invest. 2025 Aug 12;135(20):e191773. doi: 10.1172/JCI191773 (PMC12520690; doi:10.1172/JCI191773)

Figure 2B

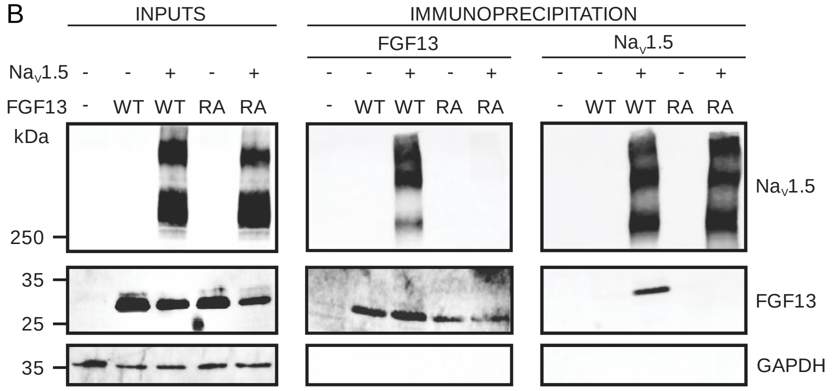
B
INPUTS
IMMUNOPRECIPITATION
FGF13
Na$_V$1.5
Na$_V$1.5 - - + - +
FGF13 - WT WT RA RA
kDa
250
35
25
35
Na$_V$1.5
FGF13
GAPDH

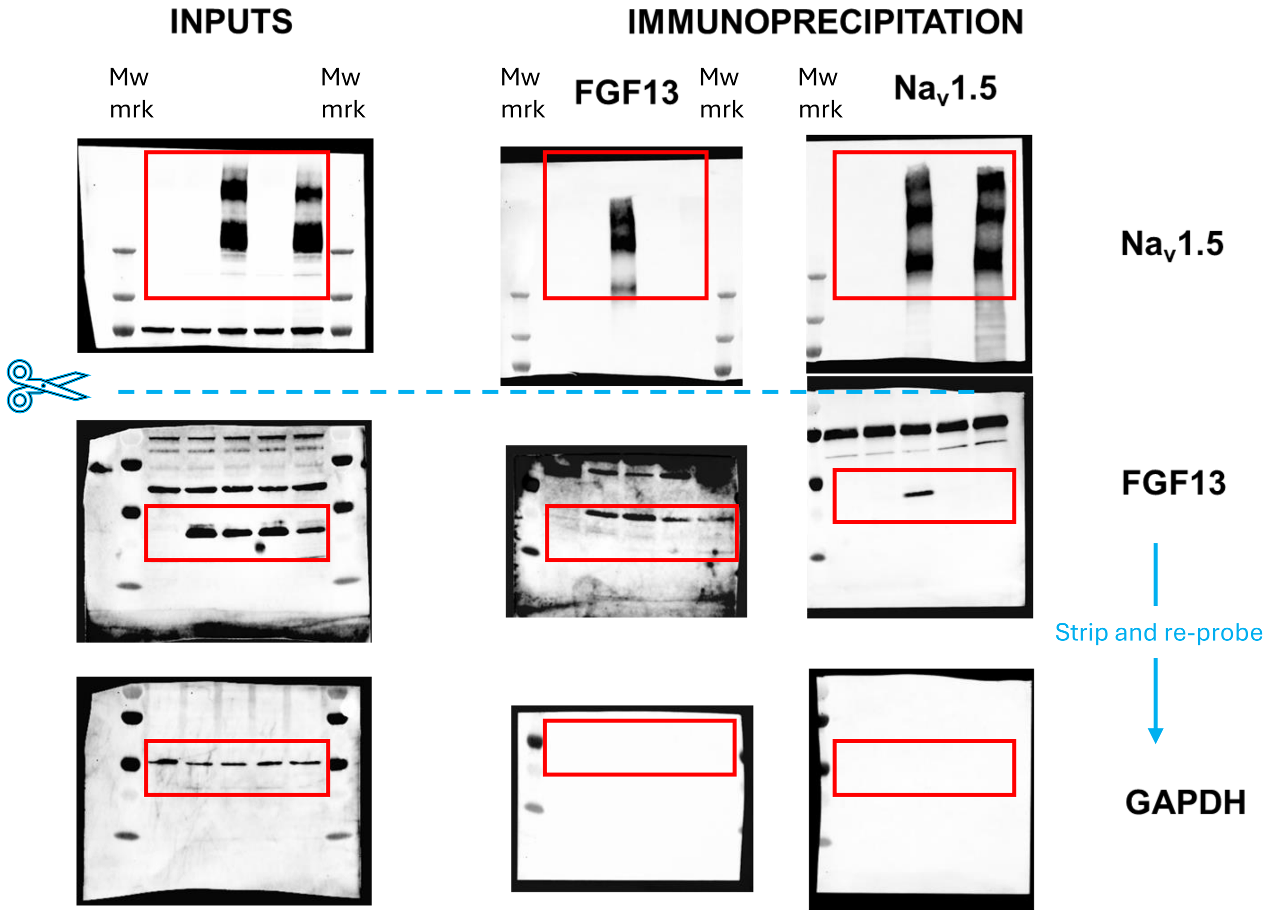
INPUTS
IMMUNOPRECIPITATION
Mw mrk
FGF13
Na$_V$1.5
Na$_V$1.5
FGF13
Strip and re-probe
GAPDH

Figure 2D

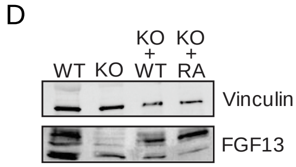

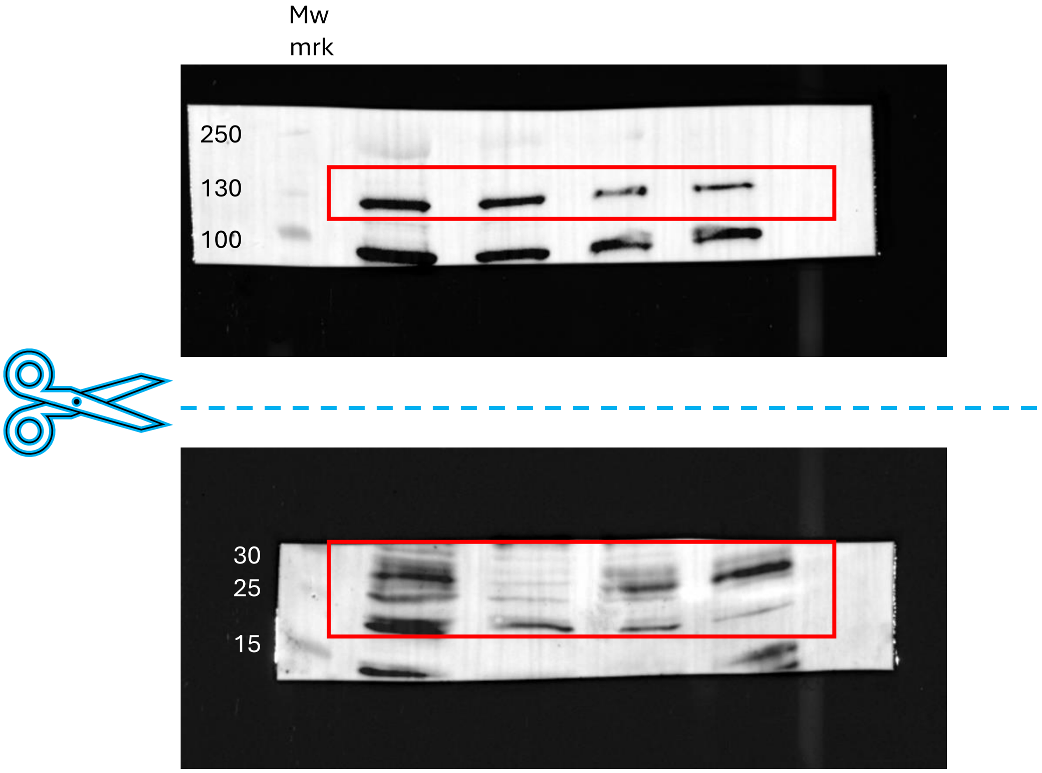

Figure 3

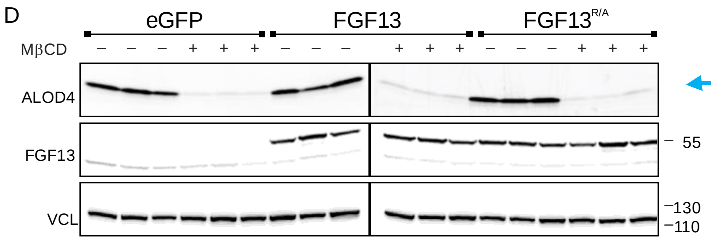

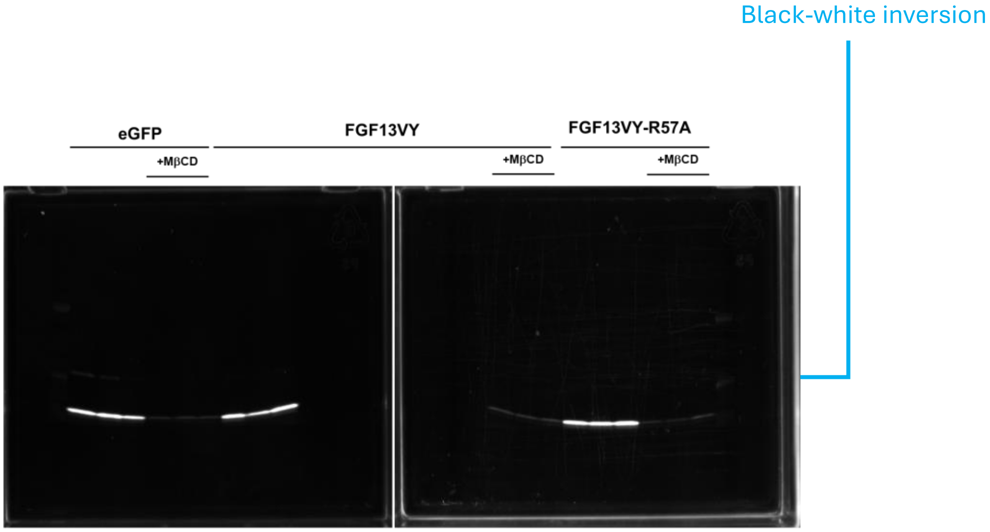

ALOD4 in gel fluorescence

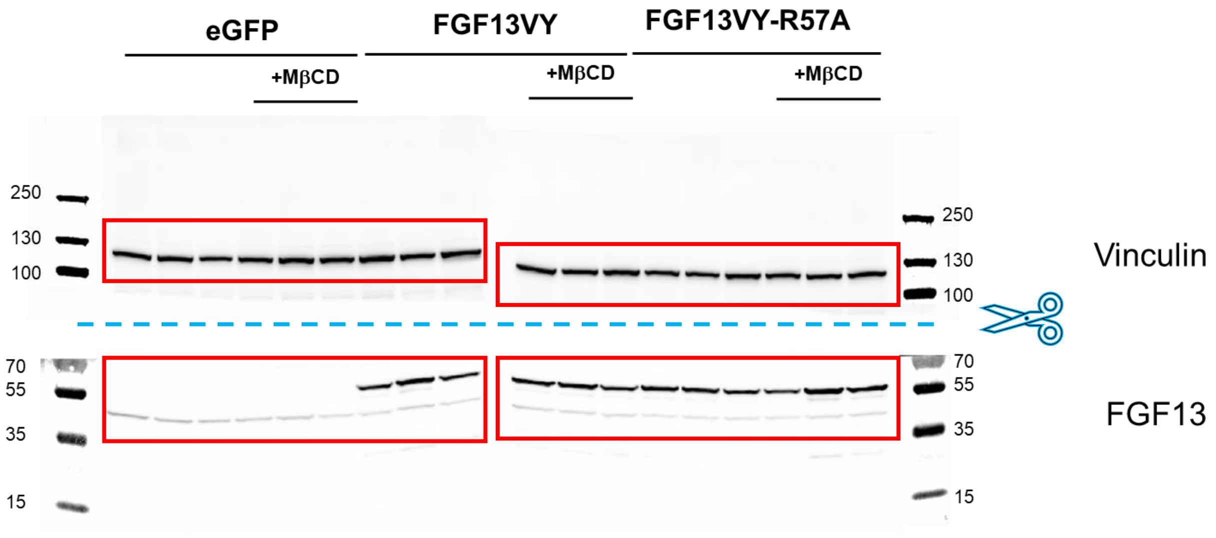

Supplement: Unedited blot and gel images [file jci-135-191773-s100.pdf]
